# Enhancement on antioxidant, anti-hyperglycemic and antibacterial activities of blackberry anthocyanins by processes optimization involving extraction and purification

**DOI:** 10.3389/fnut.2022.1007691

**Published:** 2022-10-11

**Authors:** Han Wu, Qing-Ru Di, Liang Zhong, Jian-Zhong Zhou, Cheng-Jun Shan, Xiao-Li Liu, Ai-Min Ma

**Affiliations:** ^1^College of Food Science and Technology, Huazhong Agricultural University, Wuhan, China; ^2^Institute of Agro-Product Processing, Jiangsu Academy of Agricultural Sciences, Nanjing, China; ^3^Nanjing Youwei Organic Food Company, Nanjing, China

**Keywords:** blackberry anthocyanin, enrichment, UPLC-QTOF-MS, bioactivity, nutraceutical supplement

## Abstract

This research aimed to recover anthocyanin-rich extracts from blackberry (*Rubus* spp. Hull cultivar) by optimizing the processing conditions, and to characterize anthocyanin individuals and determine influences of optimization on enhancement of antioxidant and anti-hyperglycemic activities of anthocyanins as natural supplements. The ethanol concentration of 69.87%, HCl dosage of 0.53%, solid-to-liquid ratio of 1:19.06 at 47.68°C for 17.04 h were optimal to obtain the highest extraction yield of anthocyanins at 0.72 mg/g. By using AB-8 macroporous resins, the anthocyanin concentration of 3.0 mg/mL, ethanol concentration of 90%, and elution rate of 2.0 mL/min were selected to boost the anthocyanin purity up to be 60.11%. Moreover, the purified anthocyanin extracts from blackberry contained nine main pigments which could be divided into three aglycone-based forms, and cyanidin-3-*O*-glucoside was the most abundant among them. Due to the successive processes of extraction and purification, the blackberry purified anthocyanin extracts (BA-PAE) showed much higher bioactive capacities than the blackberry crude anthocyanin extracts (BA-CAE) and blackberry fruit slurry extracts (BA-FSE), e.g., DPPH and ABTS radical scavenging activities (EC_50_ = 0.08 and 0.04, 0.32 and 0.24, and 1.31 and 0.41 mg/mL), oxygen radical absorbance capacity (1.60, 0.59, and 0.15 mmol TEAC/g), cytoprotective effects against oxidative stress in PC12 cells (1.69-, 1.58-, and 1.50-fold cell viability compared to oxidative group), α-amylase and α-glucosidase inhibitory activities (IC_50_ = 0.10 and 0.06, 0.56 and 0.32, and 3.98 and 2.16 mg/mL), and antibacterial activity (93.23, 40.85, and 80.42% reduced biofilm).

## Introduction

Blackberry (*Rubus fruticosus* L.) is popularly consumed either in fresh or processed forms. This berry provides a great many of vitamins (0.15 g/kg on average), minerals and fibers, and is also a recognized source of bioactive compounds such as polyphenols with health-related properties (1). The major phenolic compounds in blackberry fruits comprise anthocyanins, hydrolysable tannnis (ellagitannins), flavonols, and condensed flavan-3-ols (proanthocyanidins) with a degree of polymerization ranged 1 to >10 (2). Anthocyanins, pigments derived from the secondary metabolism of plants, belong to an important group of phenolic compounds within the flavonoid class. It has been reported that the different anthocyanin fractions obtained from blackberry help protect against the cardiovascular disease, cancer, inflammation, obesity, diabetes, and other chronic diseases (3). However, due to the high degree of processing, the notable loss of certain substances, such as thermosensitive and light sensitive phenolics occurred and successively generated the incorrect functioning in organisms. The industrial processing, involving steps of blanching, pasteurization, etc. decreased the content of anthocyanins in the final blackberry products (4). In this circumstance, the deficient substances mentioned above should be supplied in a variety of other forms, components of health food and dietary supplements, for example.

With the improvement of food safety requirements and the development of biology and medicine, natural compounds are gradually replacing the artificial synthetic pigments (5). In order to utilize in depth the anthocyanins from blackberry, the conventional solid-liquid extraction is usually applied among the main technologies (6). The diffusion and transformation of compounds and the simultaneous extraction of other compounds (free sugars, organic acids, amino acids and proteins) may be generated, which makes the extraction more complex (7, 8). Therefore, the polar solvents and acidified ethanol-water mixture are used to accelerate the diffusion of anthocyanins, and the extracted fraction remains to be separated and purified for further application. As previously reported (9), the macroporous resins were of great effectiveness to adsorb the phytochemicals from plant materials. Owing to the unique qualities of bioactive compounds, e.g., adsorption ability and convenience of desorption, the separation using AB-8 macroporous resins is an effective technique for purifying anthocyanins from crude blackberry extracts (7). Optimization of the conditions during extraction and purification could further increase the anthocyanin yield from blackberry fruits.

Due to the fact that the components of anthocyanins in blackberry cultivars grown in different regions, years and harvested at different times greatly varies, the plants show the nutrition, biological activity and physiology of dissimilarity (10, 11). Currently, there is a scarcity of research focusing on optimizing both the extraction and purification, and meanwhile characterizing anthocyanins related to blackberry cultivar “Hull.” Most of studies (7) concern on either single extraction or purification of anthocyanins from the specific variety of blackberry and some of other berry species. On this occasion, the current study aimed to: (i) achieve the optimum conditions for extracting anthocyanins from “Hull” blackberry using both single factor test and response surface methodology; (ii) obtain the highest yield of anthocyanins through optimizing the purification using AB-8 macroporous resins, specific to crude anthocyanin extracts; (iii) characterize the specific anthocyanin composition in blackberry “Hull” by applying the ultra-high performance liquid chromatography-tandem quadrupole time of flight mass spectrometry (UPLC-QTOF-MS); (iv) validate the obvious effects of the corresponding processes including extraction and purification on enhancement of antioxidant, anti-hyperglycemic, and antibacterial activities of blackberry anthocyanins. This research helped provide an optimized methodology for efficiently preparing anthocyanins from blackberry cultivar “Hull,” and furthermore, quantitatively took into account the excellent bioactivities of the obtained anthocyanin-fortified supplements.

## Materials and methods

### Materials and chemicals

Blackberry (*Rubus* spp. Hull cultivar) was provided by Nanjing Youwei Organic Food Co., Ltd. (Nanjing, China) in July 2021 and stored at −18°C. Some of the solvents, involving acetonitrile and methanol, were HPLC grade and purchased from Merck Chemicals Co., Ltd. (Darmstadt, Germany). The cyanidin-3-glucoside (C3G) was obtained from Sigma-Aldrich Co., Ltd. (St. Louis, USA), and the other chemicals were from Sinopharm Chemical Reagent Co., Ltd. (Beijing, China). A rat adrenal medulla pheochromocytoma differentiated cell (PC12) line was obtained from Taide Biological Technology (Nanjing, China). The dulbecco's modified eagle medium (DMEM) and fetal bovine serum (FBS) were provided by Gibco/Invitrogen (Shanghai, China), and the kits of MTT cell proliferation and cytotoxicity assay and reactive oxygen species (ROS) assay were from Beyotime Biotechnology (Shanghai, China).

### Extraction of blackberry anthocyanins

#### Extraction optimized by single-factor analysis

After defrosting blackberry fruits, the anthocyanins were extracted using an acidifying ethanol solution (8). Specifically, the blackberry was added with distilled water at a series of solid-liquid ratios 1:3, 1:5, 1:10, 1:15, 1:20, and 1:25 (w/v) and homogenized for 30 s (80% ethanol, 0.25% HCl for 12 h at 40°C). Then the fruit slurry was soaked in the ethanol (50, 60, 70, 80, 90, 100%, v/v) containing hydrochloric acid (0.05, 0.1, 0.25, 0.5, 0.75, 1%, v/v) (0.25% HCl or 80% ethanol, solid-liquid ratios of 1:15 for 12 h at 40°C). The durations were of 1, 3, 6, 12, 18, and 24 h (80% ethanol, 0.5% HCl, solid-liquid ratios of 1:15 at 40°C), and extraction temperatures were chosen of 20, 30, 40, 50, 60°C (80% ethanol, 0.5% HCl, solid-liquid ratios of 1:15 for 18 h). The supernatant collected after centrifugation at 5,000 g for 15 min was used for determining the yield of anthocyanins.

#### Extraction optimized by response surface methodology

The influences of multi-factors and those of their interactions on yield of blackberry anthocyanins was evaluated by employing the Box-Behnken design (BBD) in response surface methodology (12). The five factors were selected as ethanol concentration (A), hydrochloric acid concentration (B), extraction time (C), extraction temperature (D), and solid-to-liquid ratio (E). The BBD matrix was present in Table 1.

**Table 1:** Levels and codes of factors for Box-Behnken experimental design.

**Factor**		**Coding level**		
		**-1**	**0**	**1**
Ethanol concentration (%)	A	60	80	100
HCl dosage (%)	B	0.15	0.50	0.85
Extraction time (h)	C	12	18	24
Extraction temperature (°C)	D	30	50	70
Solid-to-liquid ratio	E	1:10	1:15	1:20

### Purification of blackberry anthocyanins

#### Static adsorption and desorption of AB-8 macroporous resins

A 5.0 g of activated AB-8 resins were taken into 50 mL of blackberry crude anthocyanin extracts, and the mixture was shaken at 150 rpm, 25°C for 24 h. After the above adsorption, the resins were rinsed with distilled water. The blackberry anthocyanins were subsequently desorbed by 50 mL 100% ethanol in a shaker at 150 rpm, 25°C for 24 h (13). The properties of resins were determined as follows:


(1) $$\begin{array}{l} \text{Adsortion quantity }\left(\text{mg}/\text{g}\right)=\left({\text{C}}_{0}\times {\text{V}}_{0}-{\text{C}}_{\text{a}}\times {\text{V}}_{1}\right)/\text{m} \end{array}$$

(2) $$\begin{array}{l} \text{Desorption rate}(\%)=\;\left[\;{\text{V}}_{2}\times {\text{C}}_{\text{d}}/(\text{m}\times \text{Q})\right]\;\times 100 \end{array}$$

where Q was the adsorption quantity; C_0_, C_a_ and C_d_ were the anthocyanin concentration of initial sample, surplus sample after adsorption, and desorbed solution, respectively (mg/mL); V_0_, V_1_, and V_2_ were the volumes of initial sample, surplus sample after adsorption, and desorbed solution, respectively (mL); m was the weight of resins (g).

#### Purification optimized by single-factor analysis

The influences of three factors, including anthocyanin concentration (1.0, 2.0, 3.0, 4.0, 5.0, 6.0, 7.0, and 8.0 mg/mL), ethanol concentration (0, 10, 20, 30, 40, 50, 60, 70, 80, 90, and 100%) (3.0 mg/mL of anthocyanin, 4.0 mL/min), and flow rate for desorption (0.5, 2.0, 4.0, and 6.0 mL/min) (3.0 mg/mL of anthocyanin, 90% ethanol), on efficiency of purification for blackberry anthocyanins were assessed.

### Determination of the total anthocyanin content

The TAC was determined by the spectrophotometric pH differential method (14). The potassium chloride buffer (pH 1.0) and sodium acetate buffer (pH 4.5) were added to dilute each sample, respectively, and the absorbance was measured at both 520 nm and 700 nm on a UV-P5 visible spectrophotometer (Shanghai Mapada Instruments Co., Ltd., China). The cyanidin-3-*O*-glucoside (C3G) was taken as a standard in the formula as follows:


(3) $$\begin{array}{l} \text{Total content of anthocyanin}(\text{mg}/\text{g})=\;\Delta \text{A}\times \text{M}\times \text{f}\times \text{V}/(\varepsilon \times \text{m}\times \text{L}) \end{array}$$

where ΔA = (A_520nm_-A_700nm_) at pH 1.0–(A_520nm_-A_700nm_) at pH 4.5; M was the molecular weight of C3G (449.2 g/mol); f was the dilution fold of sample; V was the sample volume (mL); ε was the molar extinction coefficient of C3G (26,900 L/mol·cm); m was the sample weight (g); L was the cuvette length (cm).

### Characterization of blackberry anthocyanins using UPLC-QTOF-MS

The purified blackberry anthocyanins were qualitatively analyzed by UPLC-QTOF-MS system equipped with C18 column (250 × 4.6 mm, 5 μm particle size; G2-XS QTOF, Waters Corp., USA). The flow rate was 0.35 mL/min, and the mobile phase A consisted of 0.1% formic acid in water and mobile phase B contained 0.1% formic acid in acetonitrile. The gradient elution was 5% B for 0.5 min, 5–40% B over 20 min, and 40–95% B over 2 min, and stayed 95% B for 7.5 min (15). The mass spectrometry was performed using the electrospray source in positive ion mode, with a selected mass range of 50–1,200 m/z. The purity of blackberry purified anthocyanin extracts (5 mg/mL) was determined in accordance to the standard curve y = 2.0691 x + 10.075 (*R*^2^ = 0.9993) with C3G as a standard.

### Determination of antioxidant activities of blackberry anthocyanins

#### DPPH radical scavenging activity

The different samples, namely blackberry purified anthocyanin extracts (BA-PAE), blackberry crude anthocyanin extracts (BA-CAE), and blackberry fruit slurry extracts (BA-FSE), were lyophilized for their bioactivity analysis. A 2.0 mL of each sample at different concentrations was added to 2.0 mL of DPPH• solution (0.2 mmol/L). The mixture was allowed to stand in the dark for 30 min and measured the absorbance at 517 nm (16, 17). DPPH radical scavenging activity was calculated as follows:


(4) $$\begin{array}{l} \text{DPPH radical scavenging activity}(\%)=(1-{\text{A}}_{\text{s}}/{\text{A}}_{\text{c}})\times 100 \end{array}$$

where A_s_ was the absorbance of samples and the A_c_ was that of control. The results were expressed as EC_50_ (half maximal effective concentration), i.e., mg/mL.

#### ABTS radical scavenging activity

As reported by Wu et al. (18), ABTS^+^• was generated by oxidizing ABTS (7 mmol/L) with potassium persulfate (2.5 mmol/L) for 16 h. A 1.0 mL of each sample at different concentrations was added to 4.0 mL of ABTS^+^• solution. The mixture reacted in the dark for 6 min, and its absorbance was recorded at 734 nm. ABTS radical scavenging activity was determined using the equation below:


(5) $$\begin{array}{l} \text{ABTS radical scavenging activity}(\%)=(1-{\text{A}}_{\text{s}}/{\text{A}}_{\text{c}})\times 100 \end{array}$$

where A_s_ was the absorbance of samples and the A_c_ was that of control. The results were expressed as EC_50_, i.e., mg/mL.

#### Oxygen radical absorbance capacity

The method of detecting the ORAC was as previously described at 37.0°C in pH 7.40 phosphate buffer (18, 19). One hundred microliters of each sample at different concentrations and 50 μL of 0.2 μmol/L fluorescein were mixed. After preincubation at 37°C for 15 min, 50 μL of 80 mmol/L AAPH solution was added immediately. The fluorescence was recorded by an LB 941 TriStar Microplate Reader (Berthold Technologies, Germany) with 485-P excitation and 535-P emission filters every minute during a 100 min process. The results were expressed as Trolox equivalent antioxidant capacity (TEAC), i.e., mmol TEAC/g.

#### Cytoprotection in oxidative-damaged PC12 cells

PC12 cells were cultured in DMEM supplemented with 10% FBS and 1% streptomycin and penicillin at 37°C in a 5% CO_2_ MIR-254 humidified incubator (Sanyo Denki, China) (20). The cells were first treated with each sample at different concentrations (1, 5 and 10 μg/mL) for 12 h. A 250 μmol/L corticosterone (CORT) was then added to induce oxidative stress for another 12 h. A normal cell group with neither sample nor CORT was used as the control, and a group of CORT-induced cells was as the oxidative model. The cell viability was determined by MTT method (21). Detailly, the treated cells were cultured with 20 μL of 5 mg/mL MTT for 4 h, and the formazan crystals were dissolved by adding 150 μL of dimethyl sulfoxide (DMSO). After the absorbance measurement at 490 nm, the cell viability levels were calculated as below:


(6) $$\text{Viability level }({\text{fold increase})\;=\text{ A}}_{\text{s}}{\;/\text{ A}}_{\text{c}}\;$$

where A_s_ was the absorbance of oxidative-model of sample group, and A_c_ was that of control group. Moreover, a dichlorodihydrofluorescein diacetate (DCFH-DA) detection kit was used to evaluate ROS level in PC12 cells (22). The cultured cells were washed with phosphate buffered saline (PBS), and 0.01 mmol/L DCFH-DA was added to start the reaction for 37 °C for 20 min. After being washed thoroughly with PBS to remove the DCFH-DA that did not enter cells, the cells were collected and suspended, and seeded in the 12-well plate. The fluorescence was immediately observed under a U-RFL-T inverted microscope (Olympus, Japan) with 488 nm and 525 nm for excitation and emission filters.

### Determination of anti-hyperglycemic activities of blackberry anthocyanins

#### α-amylase inhibitory activity

A 500 μL of each sample (BA-PAE, BA-CAE, and BA-FSE) was prepared at different concentrations using sodium phosphate buffer (pH 6.9), followed by adding 500 μL of α-amylase solution (0.5 mg/mL). The mixture was incubated at 37°C for 10 min, and then 500 μL of the substrate (1% starch) was added. After the second incubation using the above conditions, the reaction was stopped by adding 1.0 mL of 3, 5-dinitrosalicylic acid and incubated at 100°C for 5 min (23). The absorbance was measured at 540 nm, and α-amylase inhibitory activity was determined as follows:


(7) $$\begin{array}{l} \;\alpha -\text{Amylase inhibitory activity}(\%)=(1-{\text{A}}_{\text{s}}/{\text{A}}_{\text{c}})\times 100 \end{array}$$

where A_s_ was the absorbance of samples and the A_c_ was that of control. The results were expressed as IC_50_ (half maximal inhibitory concentration), i.e., mg/mL (24).

#### α-glucosidase inhibitory activity

A 50 μL of each sample diluted to be different concentrations was added with 100 μL of α-glucosidase solution (0.5 U/mL) prior to incubation at 37°C for 10 min. After that, 50 μL of pNPG (5 mmol/L) was added and the mixture was incubated again at 37 °C for 5 min. The reaction was stopped by adding 80 μL of sodium carbonate (0.2 mol/L) (23, 25). The absorbance was measured at 405 nm. The equation shown below was used to calculated α-glucosidase inhibitory activity.


(8) $$\begin{array}{l} \;\alpha -\text{Glucosidase inhibitory activity}(\%)=(1-{\text{A}}_{\text{s}}/{\text{A}}_{\text{c}})\times 100 \end{array}$$

where A_s_ was the absorbance of samples and the A_c_ was that of control. The results were expressed as IC_50_, i.e., mg/mL.

### Determination of antibacterial activity of blackberry anthocyanins

#### Preparation of bacterium

The bacterium *Listeria monocytogenes* (21,532) was provided by China Center of Industrial Culture Collection (CICC) and conserved in Jiangsu Academy of Agricultural Sciences, China. It was prepared for two successive transfers in Brain Heart Infusion (BHI) broth for 24 and 12 h, respectively at 37°C before use.

#### Anti-biofilm activity

Briefly, the tested bacterium (1.0%, v/v, 0.6 OD_600nm_) companied with samples (BA-PAE, BA-CAE, and BA-FSE) of various concentrations (0.2, 0.5, 1.0, 2.0, and 5.0 mg/mL) was inoculated at 37 °C for 24 h. As to quantify the biofilm, the mixed culture needed to be washed by sterilized saline, stabilized by methyl alcohol, and stained with 0.1% crystal violet. After removing the excess dye, 95% ethanol was used to dissolve the dye adhered to biofilm (26). The absorbance was measured at 595 nm, and the computational formula was used as follows:


(9) $$\begin{array}{l} \text{Biofilm inhibition}(\%)=(1-{\text{A}}_{\text{s}}/{\text{A}}_{\text{c}})\times 100 \end{array}$$

where A_s_ was the absorbance of samples and the A_c_ was that of control. The results of anti-biofilm activity could directly indicate that of antibacterial activity of blackberry anthocyanins.

### Statistical analysis

All the data were expressed as mean ± standard deviation (SD) of more than three independent experiments. OriginPro^®^ 2021 (OriginLab Corp., USA) was applied to plot the figures. One-way analysis of variance (ANOVA) and Duncan's multiple comparison tests were used to determine the significant differences between means (*P* < 0.05) by using IBM SPSS Version 22.0 (SPSS Inc., USA). Design expert software version 7.0 (StaEase Corp., USA) was used to conduct the modeling and statistical analysis in response surface experiments.

## Results and discussion

### Effects of extraction conditions on yield of blackberry anthocyanins

**Figure 1:**
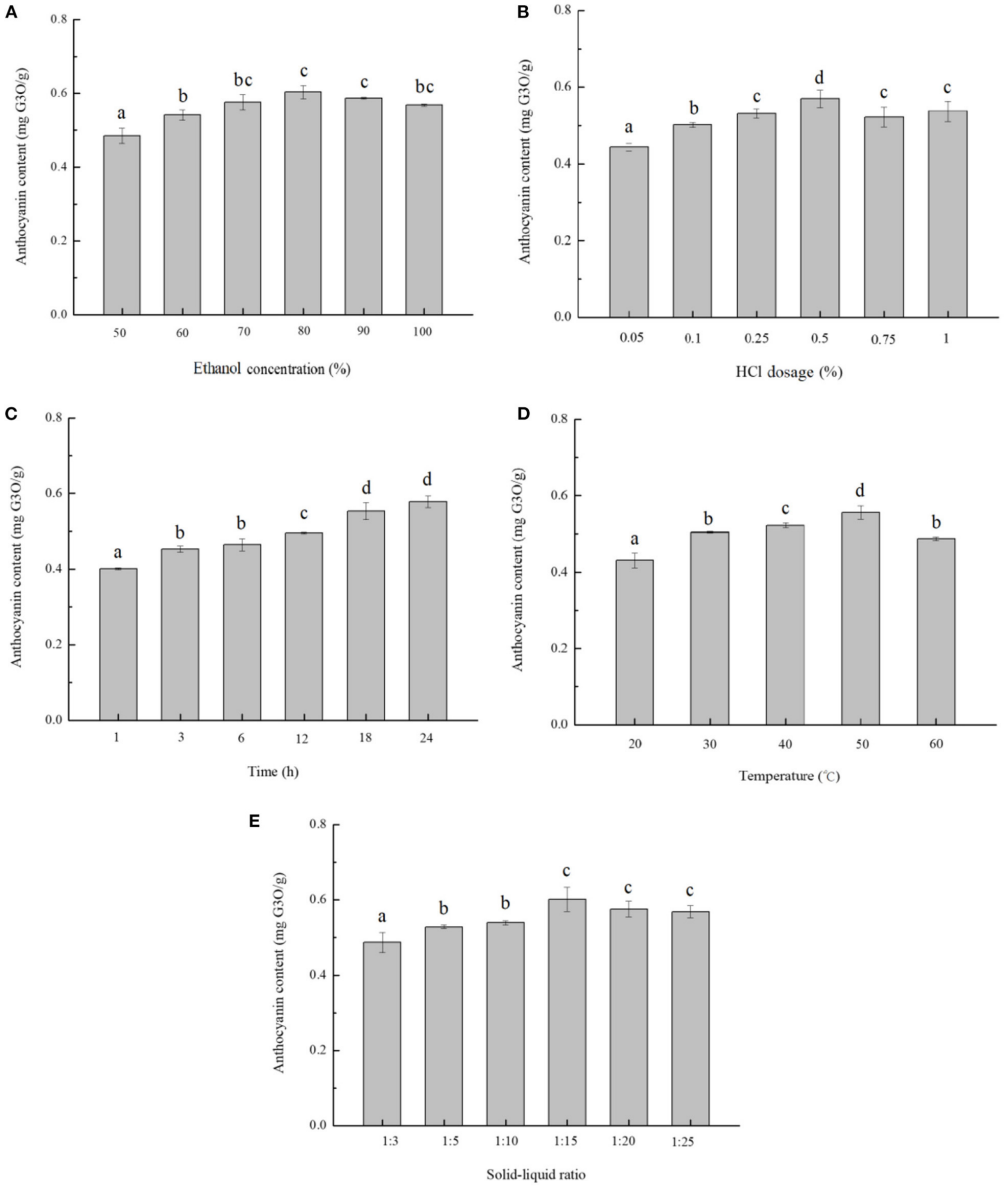
Single-factor analysis of anthocyanin extraction from blackberry. The effects of different extraction parameters: **(A)** ethanol concentration, **(B)** HCl concentration, **(C)** extraction time, **(D)** extraction temperature, and **(E)** solid-to-liquid ratio on anthocyanin extraction yield were studied. The mean values in the same figure with different lowercase letters were significantly different at the level of 0.05.

Figure 1A showed the yield of blackberry anthocyanins changed positively as the ethanol concentration increased from 50 to 80%, however, decreased slightly with the purity of ethanol over 80%. The blackberry anthocyanin output peaked at 0.61 ± 0.02 mg/g. Liu et al. (8) have reported that a raising concentration of ethanol restrained the dissolution of sugars, pectins, and other highly polar water-soluble pigments, and enhanced the yield of anthocyanins. On the other hand, the polarity difference between anthocyanins and solvent was diminished and impeded the anthocyanin extraction, along with the increase in ethanol concentration. Moreover, due to the stability of anthocyanin in an acidic environment, the complex bonds between anthocyanins and metal ions, the ionic bonds between polysaccharides and proteins, and some hydrogen bonds could be broken (27). This phenomenon resulted in the release of anthocyanins and increased their extraction efficiency. As shown in Figure 1B, by adding 0.50 % of HCl, the anthocyanin yield arrived at the maximum as 0.57 ± 0.02 mg/g. However, the excessive acids caused the partial hydrolysis of glycosidic bonds and acyl groups of anthocyanins and retarded the extraction progress (28).

In Figure 1C, the extraction yield of anthocyanins increased steadily during the starting 18 h and arrived at a maximum value of 0.55 ± 0.02 mg/g, upon which point the substances in and out of solution reached equilibrium. In terms of extraction temperature, the rate of molecular thermal mobility increased as the temperature raised, and then the solvent diffusion rate and speed of penetration were accelerated (29). The yield of anthocyanins increased with temperatures varying from 20 to 50°C, and the temperature of 50°C was optimal to obtain the highest extraction efficiency (0.56 ± 0.02 mg/g) (Figure 1D). In addition, as the solid-to-liquid ratio decreased to be 1:15, the extraction yield climbed to 0.60 ± 0.03 mg/g as maximum (Figure 1E). Consistently, Pinela et al. (30) reported that a larger amount of solvent could dissolve the polyphenol components more effectively in general, leading to a higher extraction yield, whereas the solubility of anthocyanin might be reduced by an excess of solvent.

### Optimization of extraction of blackberry anthocyanins using response surface methodology

According to the single-factor analysis above, the ethanol concentration (60, 80, and 100%), HCl dosage (0.15, 0.50, and 0.85%), extraction time (12, 18, and 24 h), extraction temperature (30, 50, and 70°C), and solid-to-liquid ratio (1:10, 1:15, and 1:20) were chosen for designing the RSM experiments (Table 1). With a range of substantial data, the RSM analyzed the responses of multiple factors and those of their interactions to optimize the extraction process (31). The yield of anthocyanins and different variables were found related by the following second-order polynomial equation: Y = – 3.6375 + 0.0796A + 0.8094B + 0.0080C + 0.0273D + 0.0295E + 0.0019AB + 0.0001AC – 0.0002AD – 0.0003AE – 0.0037BC – 0.0098BD + 0.0055BE + 0.00004CD – 0.00001CE + 0.0003DE. The results of this model designed by BBD, analysis of variance, and adequacy of model were then listed in Table 2. Overall, this model gave a *P*-value of < 0.0001, indicating that it was significant. The *R*^2^ coefficient, ${\text{R}}_{\text{Adj}}^{2}$ coefficient, and *P*-value of lack of fit revealed as well that this model could effectively simulate the progress of extraction.

**Table 2:** Box-Behnken design for the selected factors and analysis of variance for the fitted quadratic polynomial model.

**Source**	**Sum of squares**	**DF**	**Mean square**	***F*-value**	***P*-value**	**Significance**
Model	0.40	20	0.020	5.99	<0.0001	**
A	1.157E-008	1	1.157E-008	3.471E-006	0.9985	
B	9.996E-004	1	9.996E-004	0.30	0.5894	
C	2.533E-005	1	2.533E-005	7.600E-003	0.9313	
D	1.662E-004	1	1.662E-004	0.050	0.8253	
E	0.015	1	0.015	4.63	0.0426	*
AB	6.766E-004	1	6.766E-004	0.20	0.6567	
AC	1.664E-003	1	1.664E-003	0.50	0.4872	
AD	0.016	1	0.016	4.68	0.0417	*
AE	0.016	1	0.016	4.86	0.0383	*
BC	9.622E-004	1	9.622E-004	0.29	0.5964	
BD	0.019	1	0.019	5.60	0.0271	*
BE	1.503E-003	1	1.503E-003	0.45	0.5088	
CD	3.766E-004	1	3.766E-004	0.11	0.7399	
CE	3.960E-006	1	3.960E-006	1.188E-003	0.9726	
DE	0.015	1	0.015	4.38	0.0481	*
A^2^	0.18	1	0.18	52.78	<0.0001	**
B^2^	0.023	1	0.023	6.96	0.0150	*
C2	0.022	1	0.022	6.46	0.0186	*
D2	0.035	1	0.035	10.60	0.0036	**
E2	0.026	1	0.026	7.90	0.0102	*
Residual	0.073	22	3.333E-003			
Lack of fit	0.073	20	3.649E-003	21.92	0.0445	*
Pure error	3.330E-004	2	1.665E-004			
Corrected total	0.47	42				
	*R*^2^ = 0.8449	${\text{R}}_{\text{Adj}}^{2}$ = 0.7038				

*, ** indicated the significant differences at *P* < 0.05, *P* < 0.01.

**Figure 2:**
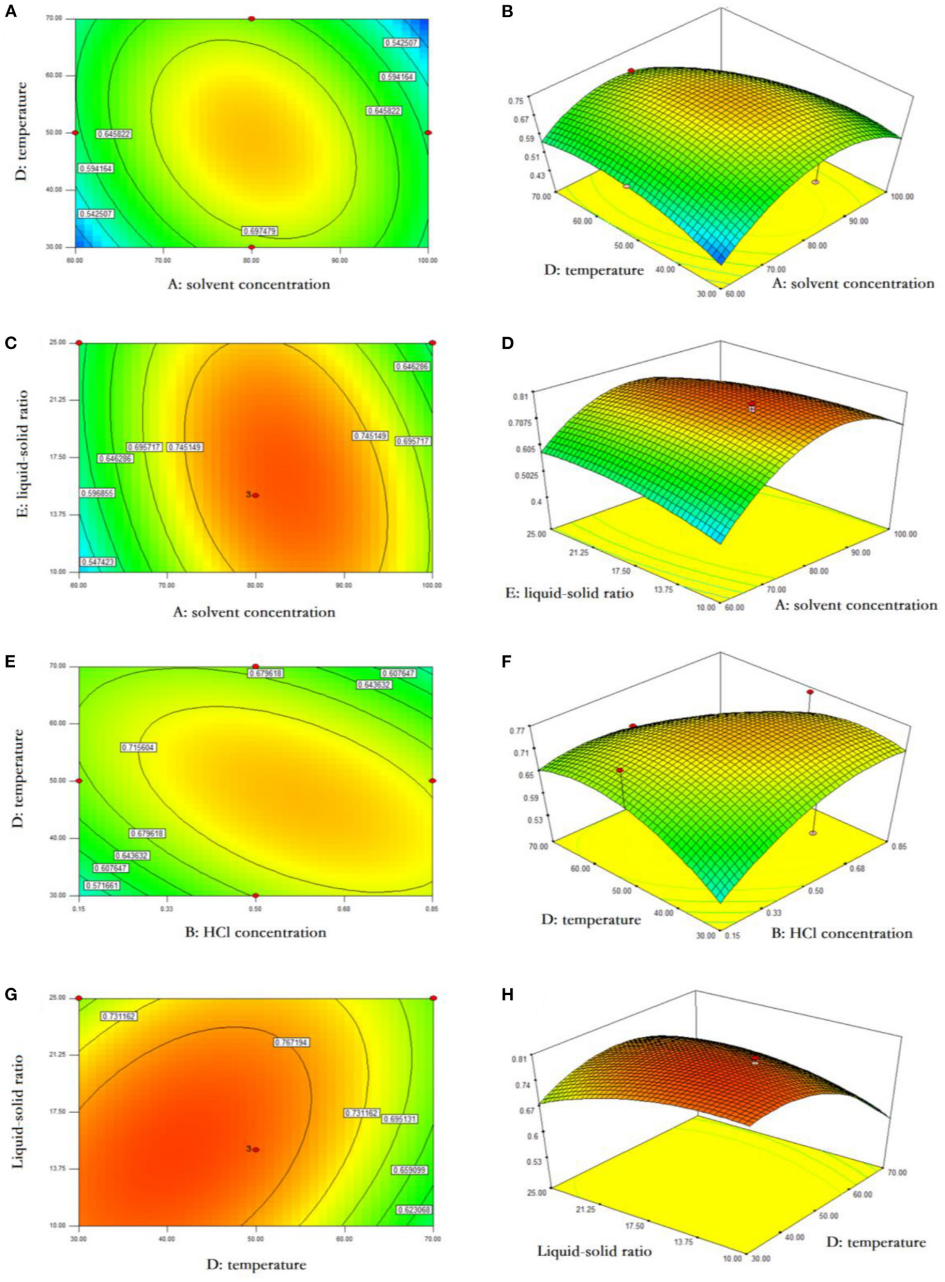
Response surface analysis of anthocyanin extraction from blackberry. The interactive effects of ethanol concentration and extraction temperature **(A,B)**, ethanol concentration and liquid-solid ratio **(C,D)**, HCl concentration and extraction temperature **(E,F)**, and extraction temperature and liquid-solid ratio **(G,H)** on anthocyanin yield were presented using the response surface plots and contour plots, respectively.

Based on F values (Table 2), the solid-to-liquid ratio was the factor most significantly affecting the yield of anthocyanins, followed by the HCl dosage, extraction temperature, extraction time, and ethanol concentration. The interactions between ethanol concentration and temperature, ethanol concentration and solid-to-liquid ratio, HCl dosage and temperature, and temperature and ratio were also shown to influence significantly the final yield of anthocyanins (*P* < 0.05) (Figure 2). As indicated by RSM results, the ethanol concentration of 69.87%, HCl dosage of 0.53%, solid-to-liquid ratio of 1:19.06 at 47.68°C for 17.04 h resulted in the largest amount of anthocyanins obtained from blackberry, which expected to be 0.73 mg/g. This finding was confirmed by an experiment conducted at above exact conditions, leading to a yield of anthocyanins at 0.72 mg/g. Similarly, Toshima et al. (11) have investigated a series of *Rubus* species and found that the anthocyanin contents of black raspberries (2.03–2.11 mg/g) were significantly higher than those of other tested species, whereas those of blackberries (“Kiowa” and “Merton Thonless”) were around 0.5 mg/g. Besides that, the genetic diversity, fertilization practices, harvest time and environmental diversity (e.g., soil type, water stress, climatic condition) were commonly considered two major factors that regulated the physicochemical and biological properties of phytochemicals in various blackberry cultivars (18).

### Optimization of purification of blackberry anthocyanins with macroporous resin column chromatography

**Figure 3:**
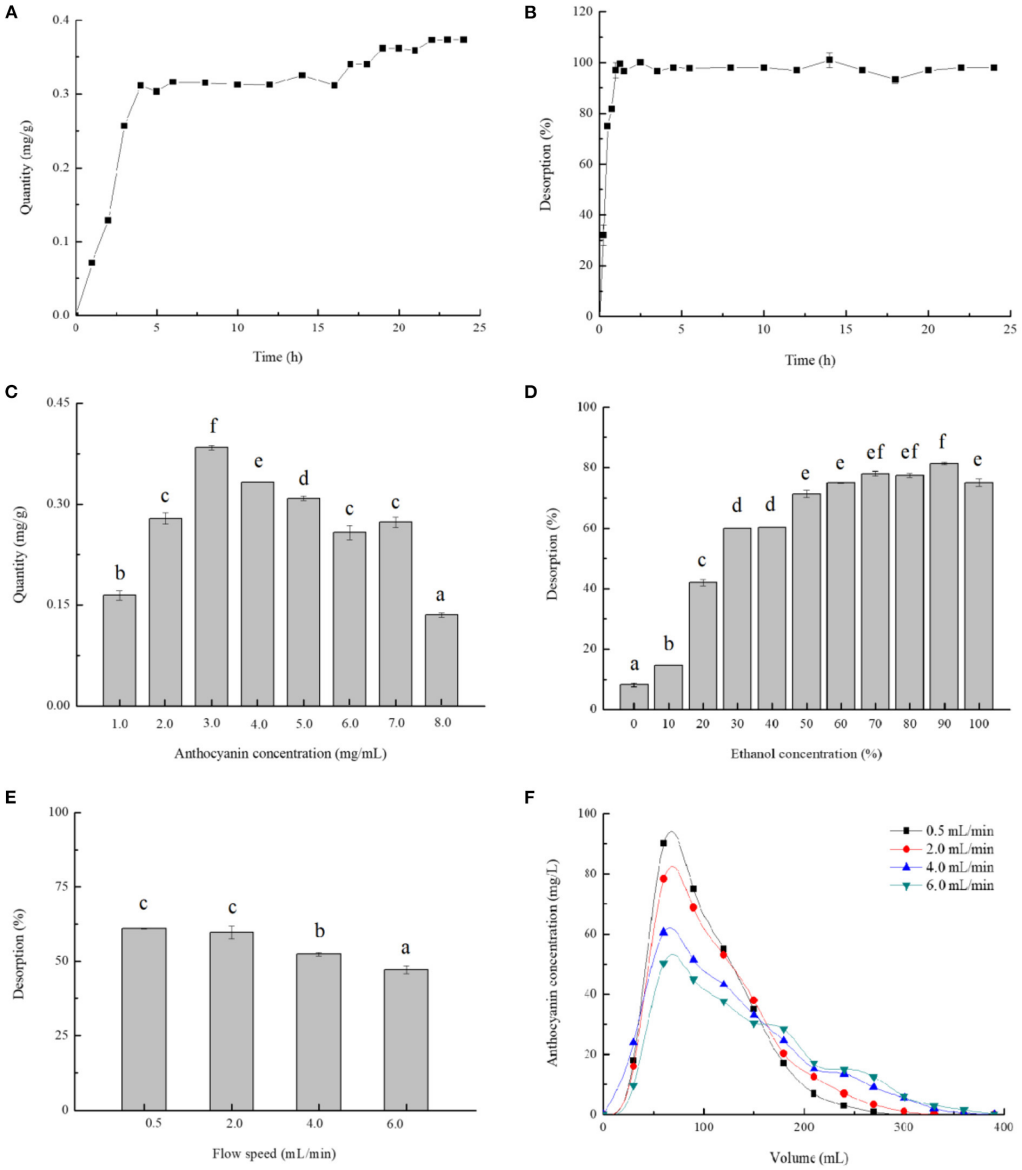
Single-factor analysis of blackberry anthocyanin purification. The adsorption kinetics **(A)** and desorption kinetics **(B)** were plotted, and the effects of different purification parameters: Anthocyanin concentration in sample **(C)**, ethanol concentration **(D)**, and flow speed **(E,F)** on absorption quantity or desorption rate were also studied. The mean values in the same figure with different lowercase letters were significantly different at the level of 0.05.

The kinetics curves relevant to adsorption and desorption of anthocyanins on AB-8 resins were separately illustrated in Figures 3A,B. During the first 5.0 h, the quantity of adsorption augmented significantly and reached 0.32 mg/g, and then remained stable and increased at a slight rate over a dozen hours. The equilibrium time for AB-8 resins desorbing blackberry anthocyanins was of 2.5 h, in which moment the desorption rate attained the maximum at 99.99%.

Furthermore, Figures 3C–F presented the effects of various parameters involving anthocyanin concentration, ethanol concentration, and flow speed on purification efficiency of anthocyanins on AB-8 resins. The adsorption quantity increased with sample concentrations changing from 1.0 to 3.0 mg/mL and showed a downward trend affected by the concentrations over 3.0 mg/mL (Figure 3C). Zhao et al. (5) have reported that the excessive anthocyanins could generate the flocculation and precipitation, resulting in the pollution and resins obstruction. Therefore, 3.0 mg/mL was indicated the most suitable sample concentration for dynamic adsorption of blackberry anthocyanins. Moreover, the ethanol could be preferably used as solvent for anthocyanin extraction from the column, because it is eco-friendly and inexpensive. The desorption rate raised with the increase of ethanol concentration and arrived at its supreme level when the ethanol concentration was of 90% (Figure 3D). As shown in Figures 3E,F, the relatively lower the flow speed (0.5 and 2.0 mL/min) was, the desorption rate of anthocyanins was higher (~60%). Consistently with Liu et al. (8), a slower elution process prolonged the time as to completely desorb materials from resins, conversely, a faster eluent flowing impacted detrimentally on their dynamic desorption.

As optimized above, the sample concentration of 3.0 mg/mL, ethanol concentration of 90%, and elution rate of 2.0 mL/min were chosen as conditions for purifying blackberry anthocyanins using macroporous resins. The purities of anthocyanin-extract powders were determined by UV-Vis spectrophotometry at 520 nm with C3G as a standard. The blackberry purified anthocyanin extracts (BA-PAE) had a purity as 60.46%, which was evidently higher than those of the blackberry crude anthocyanin extracts (BA-CAE) (11.55%) and blackberry fruit slurry extracts (BA-FSE) (3.99%), respectively.

### Qualitative and quantitative analysis of blackberry anthocyanins using UPLC-ESI-QTOF-MS

**Figure 4:**
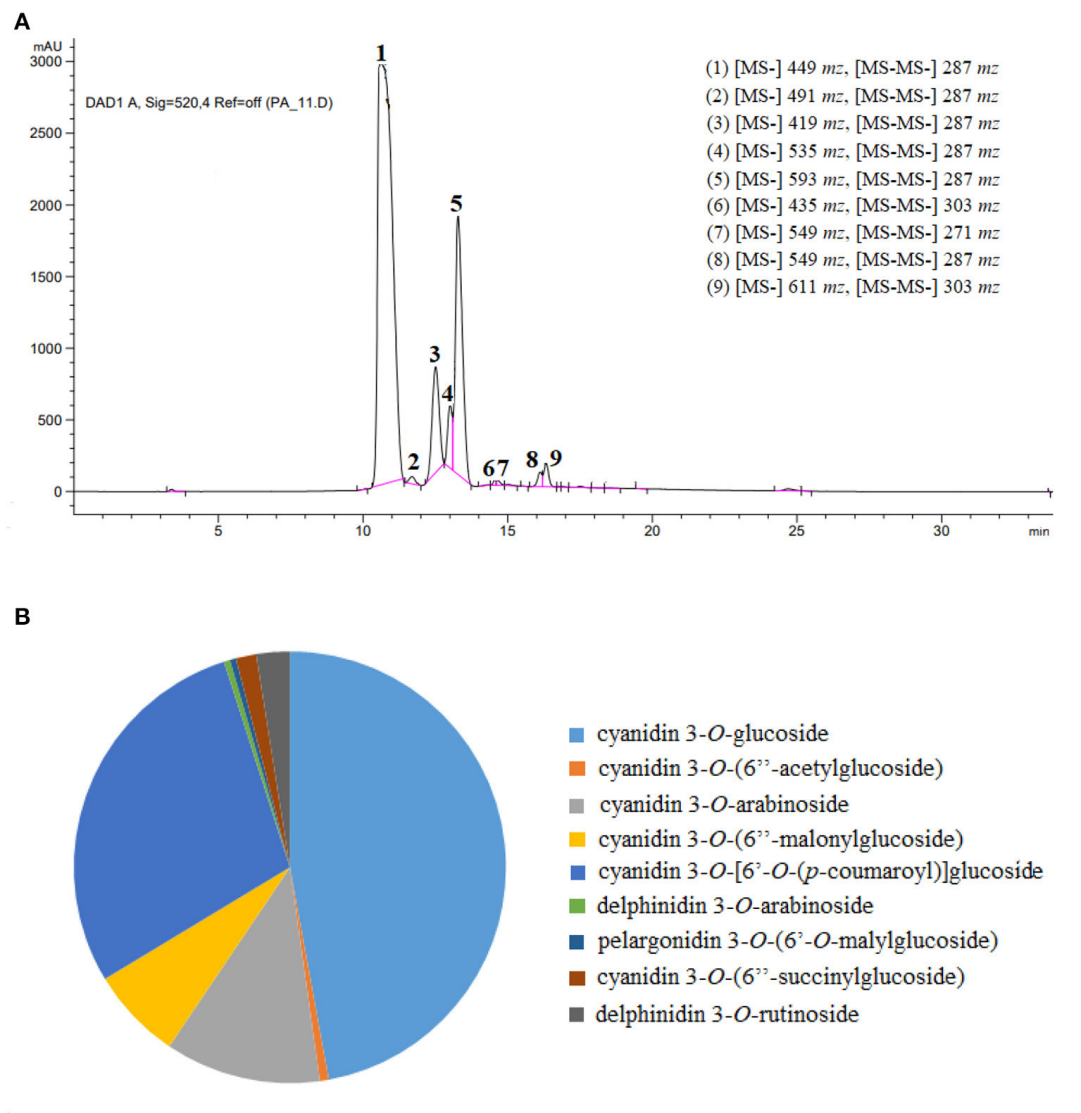
Identification of anthocyanidin compounds in blackberry purified extracts. **(A,B)** Represented the high-performance liquid chromatograms with ion fragment information, and the proportional distribution of anthocyanin individuals, respectively.

The identification of individual anthocyanins from blackberry was conducted mostly by using their elution order and retention time, and meanwhile determined by comparing their molecular and fragment ions information in UPLC-QTOF-MS with the literature data previously reported (8, 10, 32–35). The MS and MS^2^ spectra of blackberry anthocyanins were present in the Supplementary Figures 1–9. Our results indicated that there existed nine main distinct constituents in the purified anthocyanin extracts from blackberry “Hull” cultivated in Nanjing, being belong to three of the six most common types, namely cyanidin (*m/z* 287), delphinidin (*m/z* 303) and pelargonidin (*m/z* 271) (Figure 4A). Moreover, as shown in Figure 4B, the identified anthocyanins above were all quantitatively compared using cyanidin-3-*O*-glucoside as the standard. Cyanidin-3-*O*-glucoside (66.01%), cyanidin-3-*O*-[6'-*O*-(p-coumaroyl)] glucoside (19.51%), and cyanidin-3-*O*-arabinoside (7.96%) were the three substances that were the most abundant in our purified anthocyanin extracts.

Peak 1 displayed a spectral pattern [(M + H) *m/z* 449] with a molecular weight of *m/z* 287 plus *m/z* 162, and was further confirmed by the standard of cyanidin-3-*O*-glucoside in HPLC-DAD analysis. The peak 3 with [M + H] at *m/z* 419 was identified to be cyanidin (*m/z* 287) conjugated with the sugar moiety of arabinoside (*m/z* 132). Similarly, the peaks 2, 4, 5 and 8 were also identified as cyanidin derivatives, which possessed the MS/MS fragment ion of *m/z* 287 and were provisionally recognized as cyanidin-3-*O*-(6”-acetylglucoside) [(M + H) *m/z* 491], cyanidin-3-*O*-(6”-malonylglucoside) [(M + H) *m/z* 535], cyanidin-3-*O*-[6'-*O*-(p-coumaroyl)] glucoside [(M + H) *m/z* 593], cyanidin-3-*O*-(6”-succinylglucoside) [(M + H) *m/z* 549], respectively according to the previous reports (35, 36). As for peaks 6 and 9, the delphinidin aglycone was separated when the fragment ion at *m/z* 303 was generated, demonstrating that the parts of arabinoside (*m/z* 132) and rutinoside (*m/z* 308) were lost upon the fragmentation of delphinidin-3-*O*-arabinoside [(M + H) *m/z* 435] and delphinidin-3-*O*-rutinoside [(M + H) *m/z* 611], respectively. The mass spectra of peak 7, [M + H] *m/z* 549 with the molecular weight of *m/z* 271 plus *m/z* 278, demonstrated that this anthocyanin pigment might be pelargonidin-3-*O*-malyglucoside.

Other anthocyanins, such as cyanidin-3-*O*-sophoroside, cyanidin-3-*O*-rutinoside, malvidin-3-*O*-arabinoside, and malvidin-3-*O*-galactoside, were identified in other blackberries obtained in Brazil, South Africa, and Alaska (33, 37), dissimilar with those found in cultivar “Hull” used in this study. The anthocyanins from *Rubus* fruits were reported predominately cyanidin based and in the non-acylated form, and their acylated pigments were occasionally detected at low concentrations. Even so, most blackberry cultivars (Black Douglas, Chester, Evergreen, and Marion) contained the acylated individuals like cyanidin-3-malonylglucoside and cyanidin-3-dioxalyglucoside (36). Accordantly, the majority of anthocyanins (6/9) in blackberry “Hull” were based on cyanidin aglycone, among which four individuals existed in the acylated form. The pelargonidin and delphinidin based anthocyanins were also found at relatively minor levels in our blackberry, in agreement with others' reports (37, 38). The discrepancy of anthocyanin compositions among different blackberry cultivars was probably due to the differences of species, original location, preharvest climate, and other factors (10, 18).

### Antioxidant activities of blackberry anthocyanin extracts

**Figure 5:**
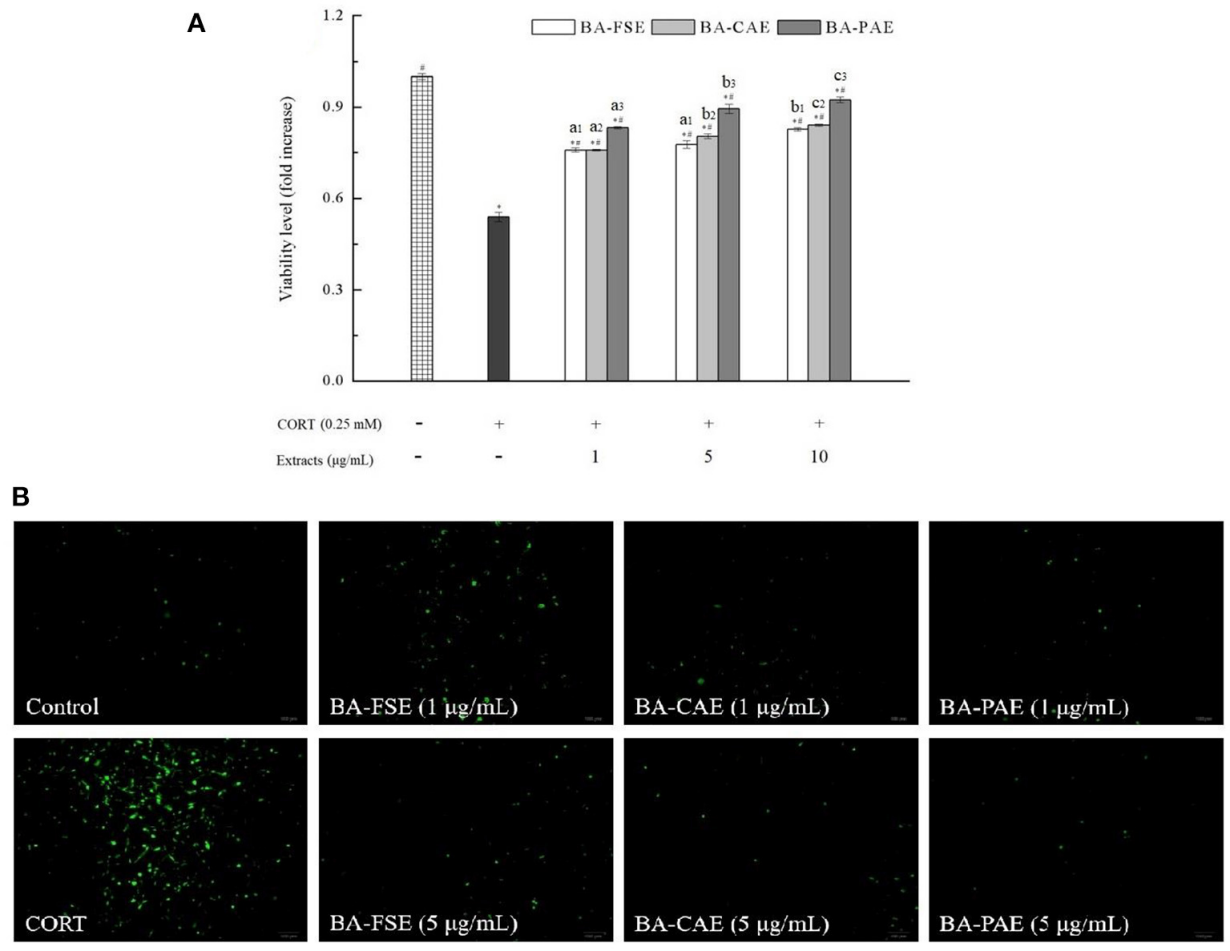
Effects of different blackberry extracts at different concentrations on cytoprotection in PC12 cells. **(A,B)** Showed the cell viability and ROS release level, respectively. * and^#^ indicated the significant differences at *P* < 0.05 between control group and others, and oxidative model group and other, respectively. Mean values of the same extract with different lowercase letters were significantly different at *P* < 0.05.

Three samples including the blackberry purified anthocyanin extracts (BA-PAE), blackberry crude anthocyanin extracts (BA-CAE), and blackberry fruit slurry extracts (BA-FSE) were lyophilized for the comparison of their different bioactivities. As mentioned above, the anthocyanin purities changed from 3.99% (BA-FSE) to 11.55% (BA-CAE), and 11.55% (BA-CAE) to 60.46% (BA-PAE) as a consequence of extraction and purification, respectively. The antioxidant activity of compounds is commonly expressed as the percentage of free radical inhibition by antioxidants, and EC_50_, the concentration required to achieve a 50% antioxidant effect, is a publicly recognized parameter for quantifying and comparing the antioxidant capacities of different antioxidants (16). Table 3 presented the DPPH and ABTS radical scavenging activities and oxygen radical absorbance capacities of different blackberry extracts. In detail, the EC_50_ of BA-PAE for DPPH• scavenging was estimated to be 0.08 ± 0.01 mg/mL and was much lower than those of BA-FSE and BA-CAE (1.31 ± 0.10 and 0.32 ± 0.03 mg/mL, respectively, *P*s < 0.05). The results of ABTS assay were in good accordance with those obtained in DPPH assay, showing that BA-PAE had the highest antioxidant capacity (EC_50_ = 0.04 ± 0.00 mg/mL), followed by BA-CAE and BA-FSE (EC_50_ = 0.24 ± 0.01 and 0.41 ± 0.01, respectively, *P*s < 0.05). As for ORAC assay, the significant differences among effective values of BA-PAE, BA-CAE and BA-FSE (1.60 ± 0.08, 0.59 ± 0.03, and 0.15 ± 0.05 mmol TEAC/g, respectively) were observed as well (*P*s < 0.05). The higher antioxidant activities of extracts rich in anthocyanins could be attributed to the optimization of extracting and purifying processes related to blackberry anthocyanins. As previously reported, the anthocyanin compounds also modify some cellular signaling processes and donate an electron/transfer hydrogen atom to free radicals, activate endogenous antioxidant mechanisms, which increases the levels of antioxidant enzymes, and act as chelators of trace metal involved in free radical protection (39). In Figure 5A, it was indicated that the blackberry extracts, namely BA-FSE, BA-CAE and BA-PAE could increase the cell viability levels from 0.53 ± 0.015-fold (vs. control group) to 0.76–0.83-, 0.76–0.84-, 0.83–0.91-fold (vs. control group) (*P* < 0.05), respectively. The effects of extracts on ROS release level (Figure 5B) in PC12 induced with CORT showed accordantly with the above results. There existed a dose-effect relationship between the extract concentrations and the ROS levels. When the concentrations were enlarged from 1 to 10 μg/mL, the production of ROS was significantly inhibited, especially in cells pretreated with BA-PAE. The reduction in ROS was relevant to the amelioration of some side effects, such as DNA mutation and genetic instability (40).

**Table 3:** Comparison of antioxidant and anti-hyperglycemic effects among the fruit slurry extract, crude anthocyanin extract, and purified anthocyanin extract.

	**BA-FSE**	**BA-CAE**	**BA-PAE**
DPPH radical scavenging activity EC_50_^1^ (mg/mL)	1.31 ± 0.10^c^	0.32 ± 0.03^b^	0.08 ± 0.01^a^
ABTS radical scavenging activity EC_50_ (mg/mL)	0.41 ± 0.01^c^	0.24 ± 0.01^b^	0.04 ± 0.00^a^
Oxygen radical absorbance capacity (mmol TEAC/g)	0.15 ± 0.05^a^	0.59 ± 0.03^b^	1.60 ± 0.08^c^
α-amylase inhibitory activity IC_50_^2^ (mg/mL)	3.98 ± 0.07^c^	0.56 ± 0.03^b^	0.10 ± 0.01^a^
α-glucosidase inhibitory activity IC_50_ (mg/mL)	2.16 ± 0.05^c^	0.32 ± 0.03^b^	0.06 ± 0.01^a^

^1^EC_50_ was the concentration for fifty percent of maximal effect of sample.

^2^IC_50_ was the half maximal inhibitory concentration of sample.

The mean values with letters a–c were significantly different at the level of 0.05.

### Anti-hyperglycemic activities of blackberry anthocyanin extracts

The carbohydrate metabolizing enzymes, α-amylase and α-glucosidase are key targets in regulating hyperglycemia. The α-amylase first catalyzes the degradation of macromolecule starch into glucose and other oligosaccharides, and the oligosaccharides are further split by α-glucosidase into monosaccharides (41). Thus, the α-amylase and α-glucosidase inhibitory activities of different blackberry extracts were measured to determine the potential relevance of extraction and purification of anthocyanins to their slowing down of the overall carbohydrate metabolism and combating to hyperglycemia (23). As shown in Table 3, BA-PAE had a significantly higher anti-hyperglycemic activity than the other extracts. The overall IC_50_ values varied from 0.10 ± 0.01 mg/mL (BA-PAE) to 0.56 ± 0.03–3.98 ± 0.07 mg/mL (BA-CAE and BA-FSE, respectively) with regard to α-amylase inhibitory activity. Similarly, the IC_50_ values for α-glucosidase inhibitory activity increased from 0.06 ± 0.01 mg/mL (BA-PAE) to 0.32 ± 0.03–2.16 ± 0.05 mg/mL (BA-CAE and BA-FSE, respectively). These results were consistent with other reports (42, 43) on raspberry and blackberry that their phenolics played a vital role in inhibiting α-amylase and α-glucosidase. In this condition, the available glucose in gut lumen was lowered, and thus the monosaccharides could hardly be absorbed and transported to blood by small intestine through sodium-glucose transporter 1 (SGLT1) and glucose transporter 2 (GLUT2) thereby increasing the postprandial blood glucose levels (44). The optimization of processing involving extraction and purification of anthocyanins could be an effective method to improve the positive effects of blackberry fruits on regulating the postprandial blood glucose level (42, 45).

### Enhancement of antimicrobial activities of blackberry anthocyanin extracts

**Figure 6:**
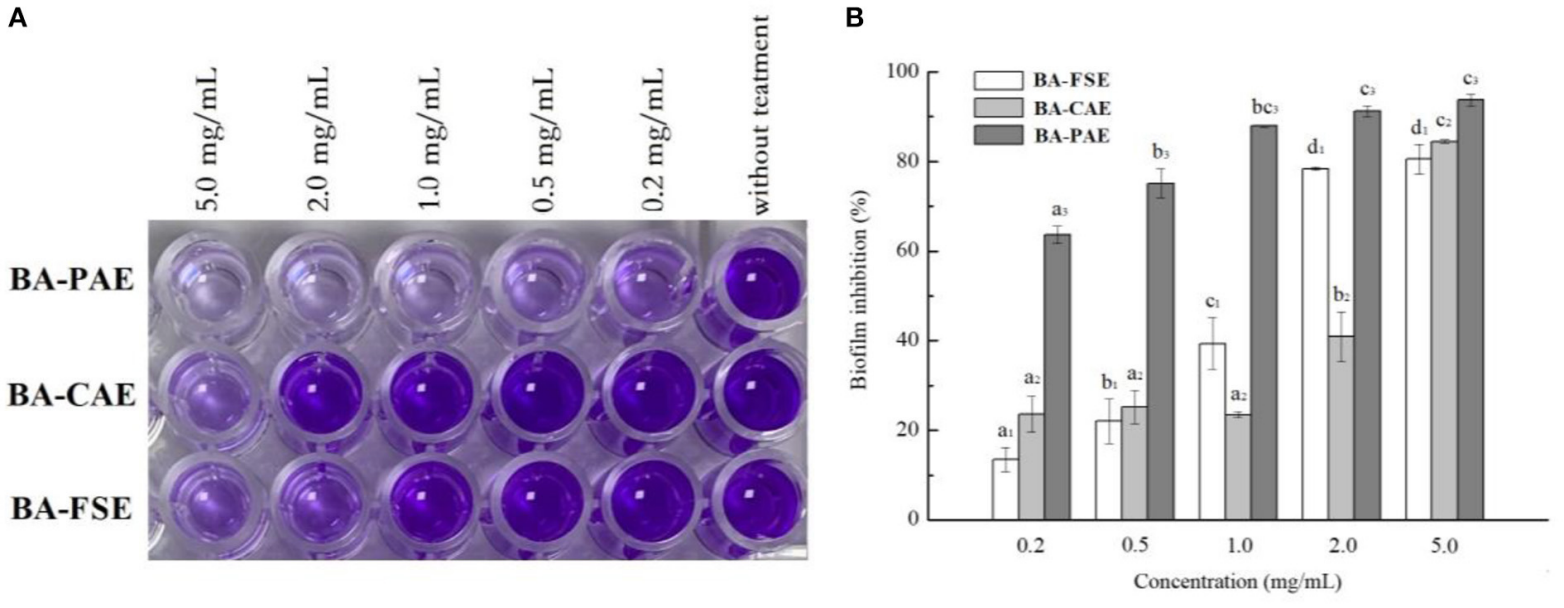
Effects of different blackberry extracts on biofilm formation in *Listeria monocytogenes*. **(A,B)** Demonstrated the results related to cell staining reaction and biofilm inhibition, respectively. The mean values for the same extract with different lowercase letters were significantly different at the level of 0.05.

The crystal violet staining method was used to quantify the *Listeria monocytogenes* biofilm changes influenced by various blackberry extracts (Figure 6). In general, the antimicrobial activities of extracts increased gradually when extracts' concentrations raised from 0.2 to 2.0 mg/mL (*P*s < 0.05). The BA-PAE showed a maximum of 93.23 ± 1.20% reduction in biofilm biomass of *Listeria monocytogenes* when its concentration was of 2.0 mg/mL, performing at a higher inhibitory rate than the BA-FSE (80.42 ± 0.39%) and BA-CAE (40.85 ± 5.50%). Consistently, Zhang et al. (26) found that the anthocyanin-rich aqueous extracts of purple highland barley bran had a high anthocyanin content and could inhibit the biofilm formation of *Pseudomonas aeruginosa* PAO1 and *Salmonella enterica* ATCC10398. In our study, some anthocyanin compounds were only detected in BA-PAE rather than in other two samples, which were cyanidin 3-*O*-(6”-malonyglucoside), cyanidin 3-*O*-(6”-succinylglucoside) and delphinidin 3-*O*-rutinoside. The cyanidin 3-*O*-[6'-*O*-(*p*-coumaroyl)] glucoside accounted for a larger percentage in BA-PAE compared to blackberry unpurified extracts. It was reported by others (8, 33, 46) that the high-purity anthocyanins could also achieve a high degree of thermal stability, and meanwhile, the anthocyanins might have greater bioactivity than the other phenolics such as condensed tannins, flavanols, flavonols and hydroxycinnamic acids existing in blackberry fruits. Therefore, the highest anthocyanin content of the purified anthocyanin extracts and the discrepancy of anthocyanin composing proportion highly contributed to their best performance of antioxidant, anti-hyperglycemic and antimicrobial activity, among different extracts. Interestingly, considering that blackberry fruits contain a wide range of other phenolic compounds and polysaccharides other than anthocyanins (13), the blackberry slurry lyophilizate possessed a better biofilm inhibitory activity than the crude anthocyanin extracts.

## Conclusions

In the current study, the solvent extraction method was used to extract anthocyanins from blackberry “Hull” grown in southeastern China, and the AB-8 macroporous resins were used for anthocyanin purification. The purity of anthocyanin-rich extracts was successfully augmented by optimizing the conditions of extraction and purification processes using single-factor analysis and response surface approach. There were nine major cyanidin-, delphinidin-, and pelargonidin-based alycone forms tentatively discovered in our blackberry anthocyanins. This finding differed slightly from those of other blackberry species grown in other regions and harvested from different origins, locations, and preharvest environmental conditions. The purified blackberry anthocyanin extracts had significantly better antioxidant, anti-hyperglycemic, and antibacterial activities than the crude blackberry anthocyanin extracts and blackberry fruit slurry extracts, indicating that the extraction and purification under optimized conditions led to an obvious enhancement on anthocyanin bioactivity. Notably, the methods used in this study were safe and efficient, and suitable for the large-scale preparation of high-purity anthocyanins from blackberry. This study provided satisfactory natural compounds that showed high potentials in food, supplements, and nutraceutical industries. In the future, the improved bioactivity of blackberry anthocyanins would be further confirmed in some of the cell and animal models. Also, our work opened the possibility to improve the functionality of designed food systems, from which the better diets based on further animal and clinical studies could be designed.

## Data availability statement

The original contributions presented in the study are included in the article/Supplementary materials, further inquiries can be directed to the corresponding author/s.

## Author contributions

Conceptualization: HW and A-MM. Data curation: HW and J-ZZ. Formal analysis and software: C-JS. Funding acquisition: LZ and X-LL. Investigation, project administration, validation, and writing—original draft: HW. Methodology: Q-RD. Resources: A-MM. Supervision: A-MM and X-LL. Visualization: Q-RD and J-ZZ. Writing—review and editing: HW and X-LL. All authors contributed to the article and approved the submitted version.

## Funding

This work was financially supported by the Science and Technology Program of Jiangsu Province under Grant BE2020380 and Jiangsu Agricultural Science and Technology Innovation Fund under Grant CX(20)3057 and Grant CX(22)2026.

## Conflict of interest

Author LZ was employed by Nanjing Youwei Organic Food Co., Ltd. The remaining authors declare that the research was conducted in the absence of any commercial or financial relationships that could be construed as a potential conflict of interest.

## Publisher's note

All claims expressed in this article are solely those of the authors and do not necessarily represent those of their affiliated organizations, or those of the publisher, the editors and the reviewers. Any product that may be evaluated in this article, or claim that may be made by its manufacturer, is not guaranteed or endorsed by the publisher.
