# Supplementary material for: Enhancement on antioxidant, anti-hyperglycemic and antibacterial activities of blackberry anthocyanins by processes optimization involving extraction and purification
Source: Front Nutr. 2022 Oct 11;9:1007691. doi: 10.3389/fnut.2022.1007691 (PMC9593095; doi:10.3389/fnut.2022.1007691)
Supplement: Supplementary file 1 [file Data_Sheet_1.pdf]

List of supplementary figures

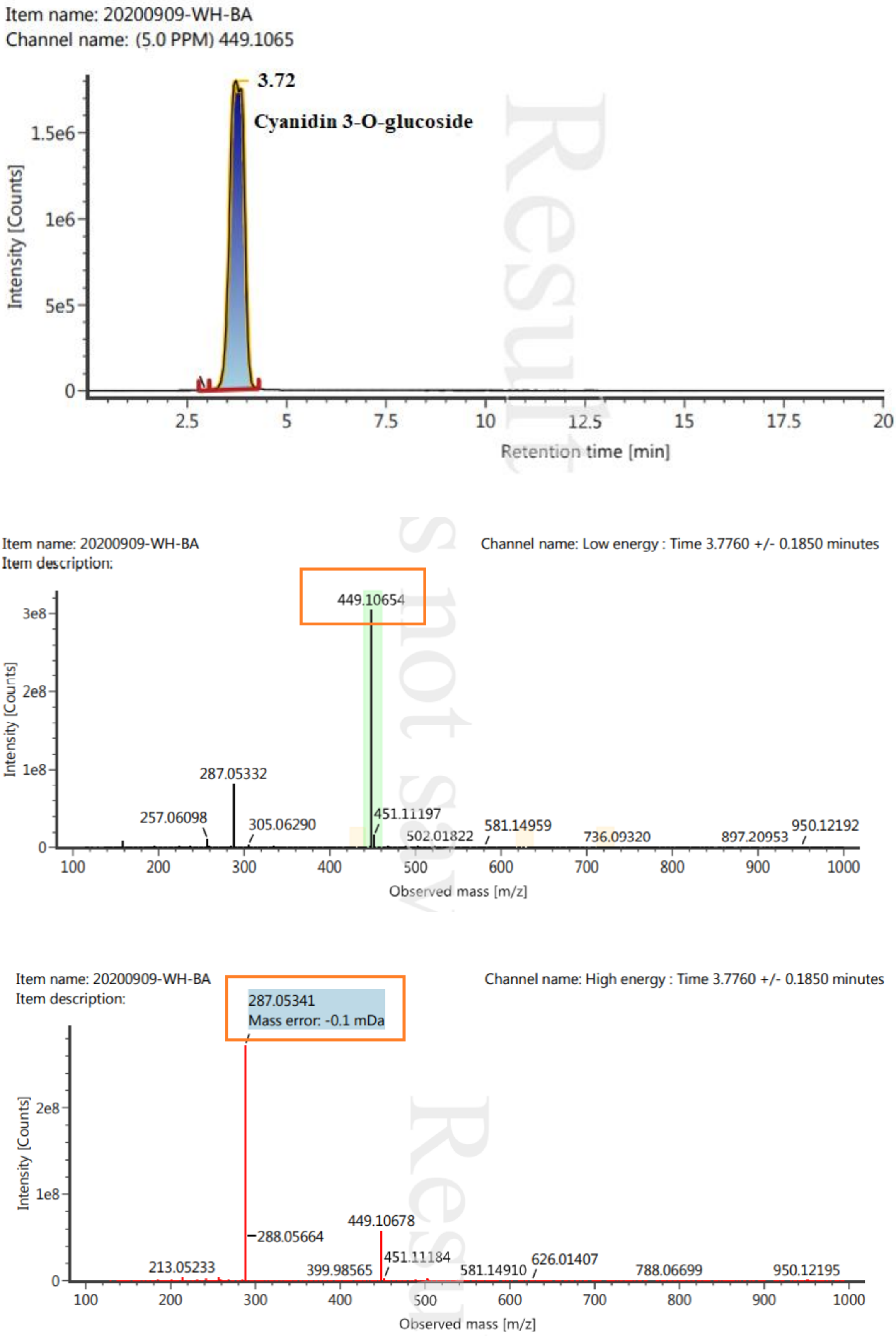

Fig. S1 MS of anthocyanin compound cyanidin 3-*O*-glucoside

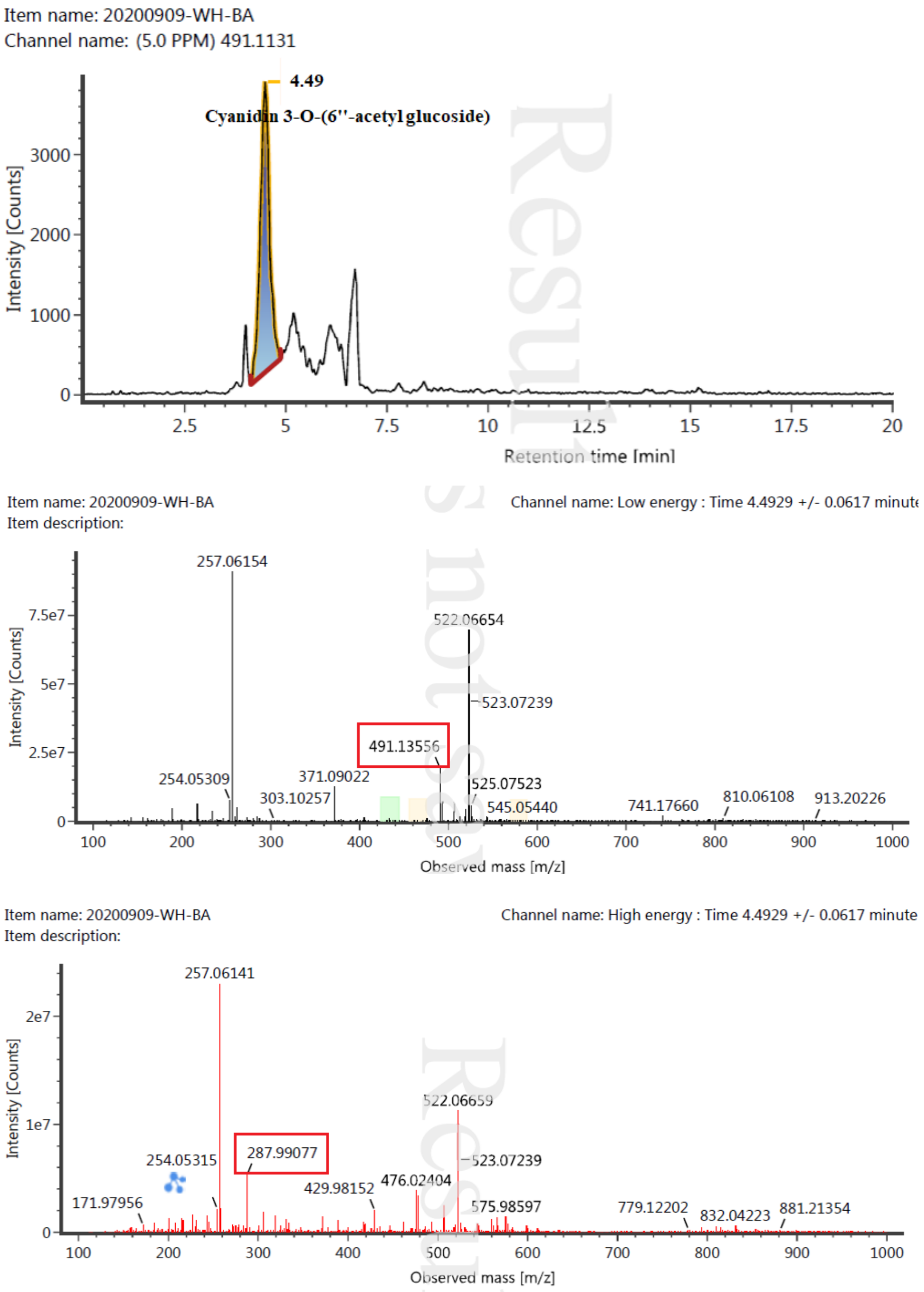

Fig. S2 MS of anthocyanin compound cyanidin 3-*O*-(6''-acetylglucoside)

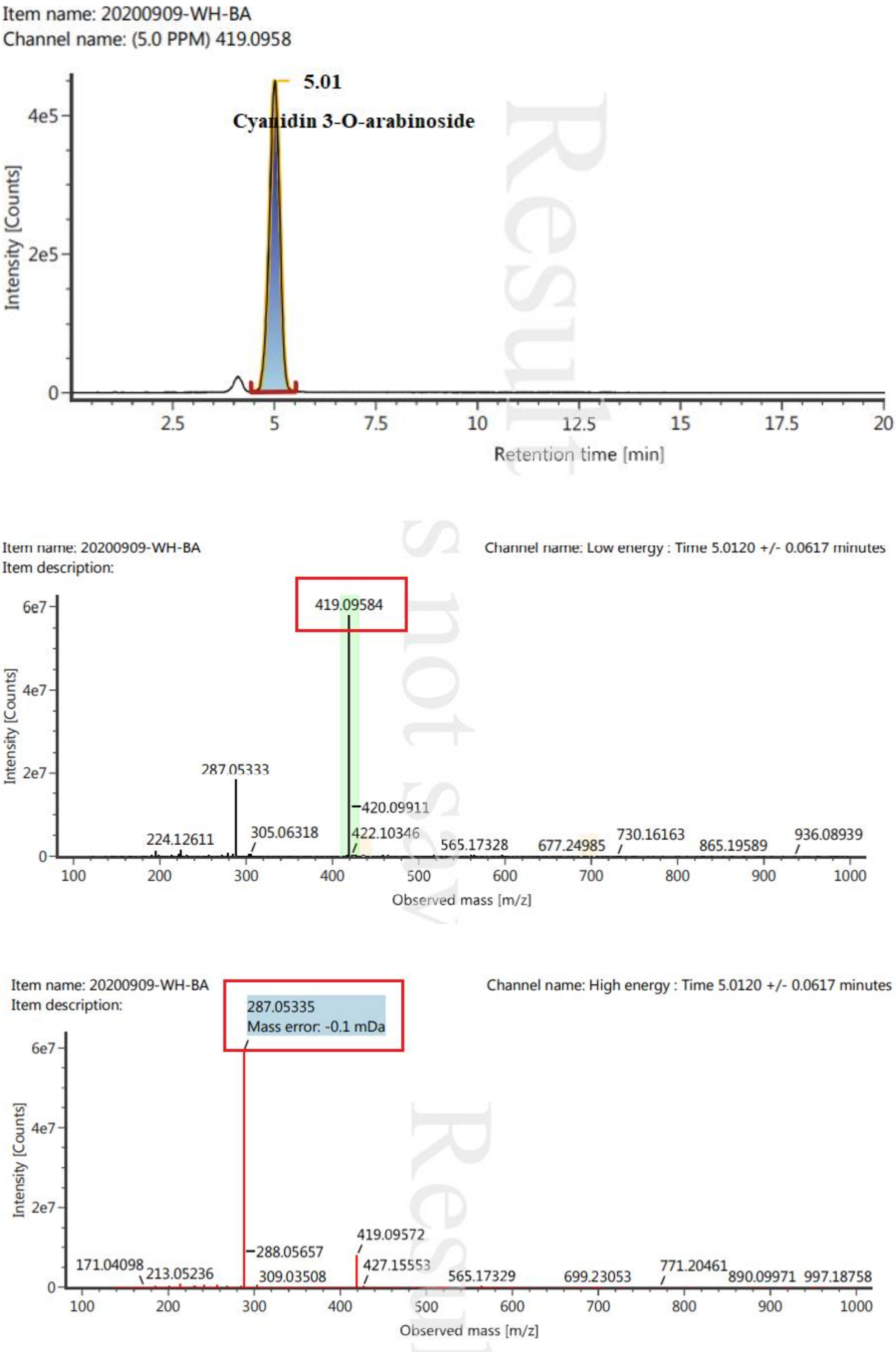

Fig. S3 MS of anthocyanin compound cyanidin 3-*O*-arabinoside

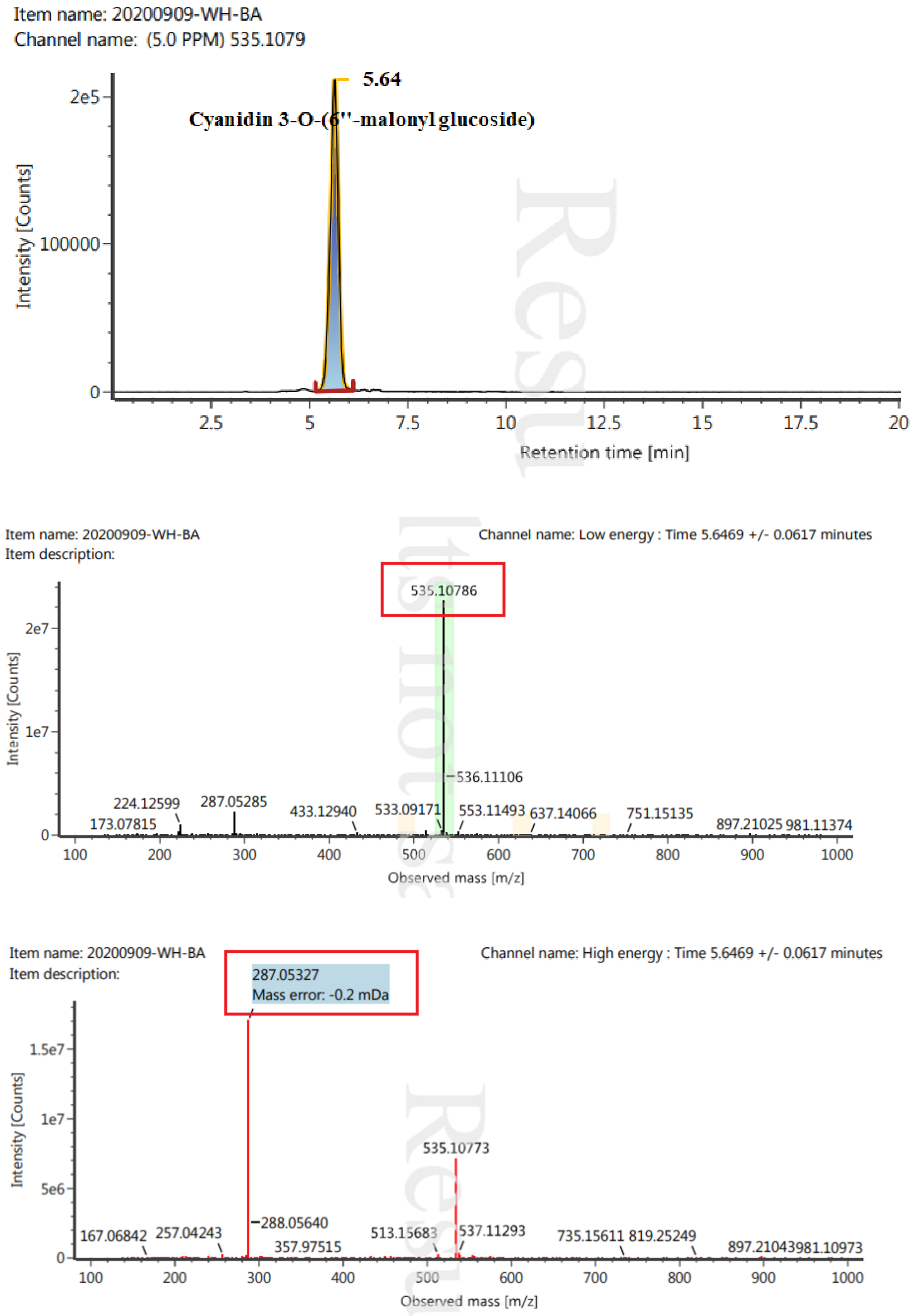

Fig. S4 MS of anthocyanin compound cyanidin 3-*O*-(6’’-malonylglucoside)

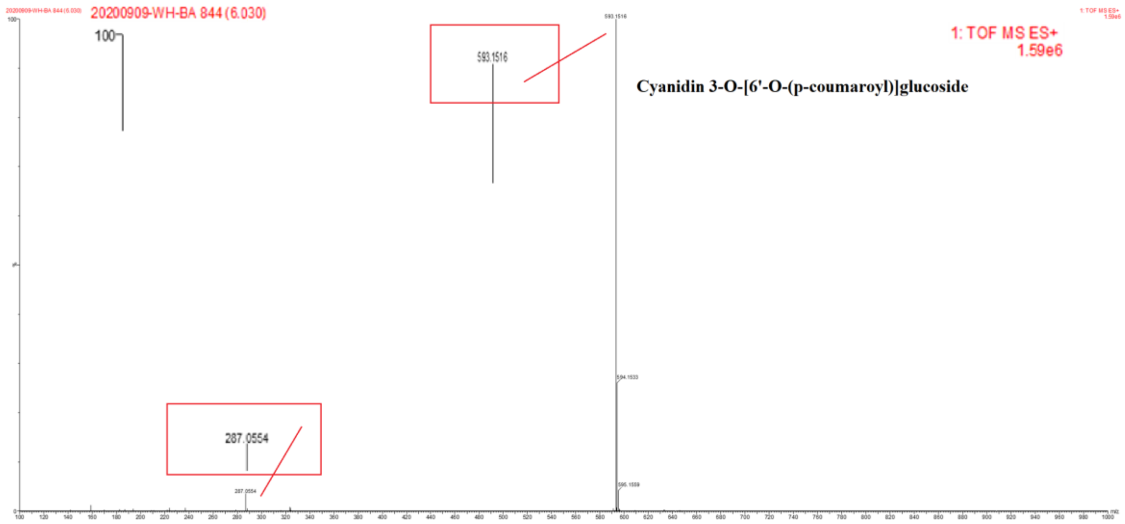

Fig. S5 MS of anthocyanidin compound cyanidin 3-*O*-[6’-*O*-(p-coumaroyl)]glucoside

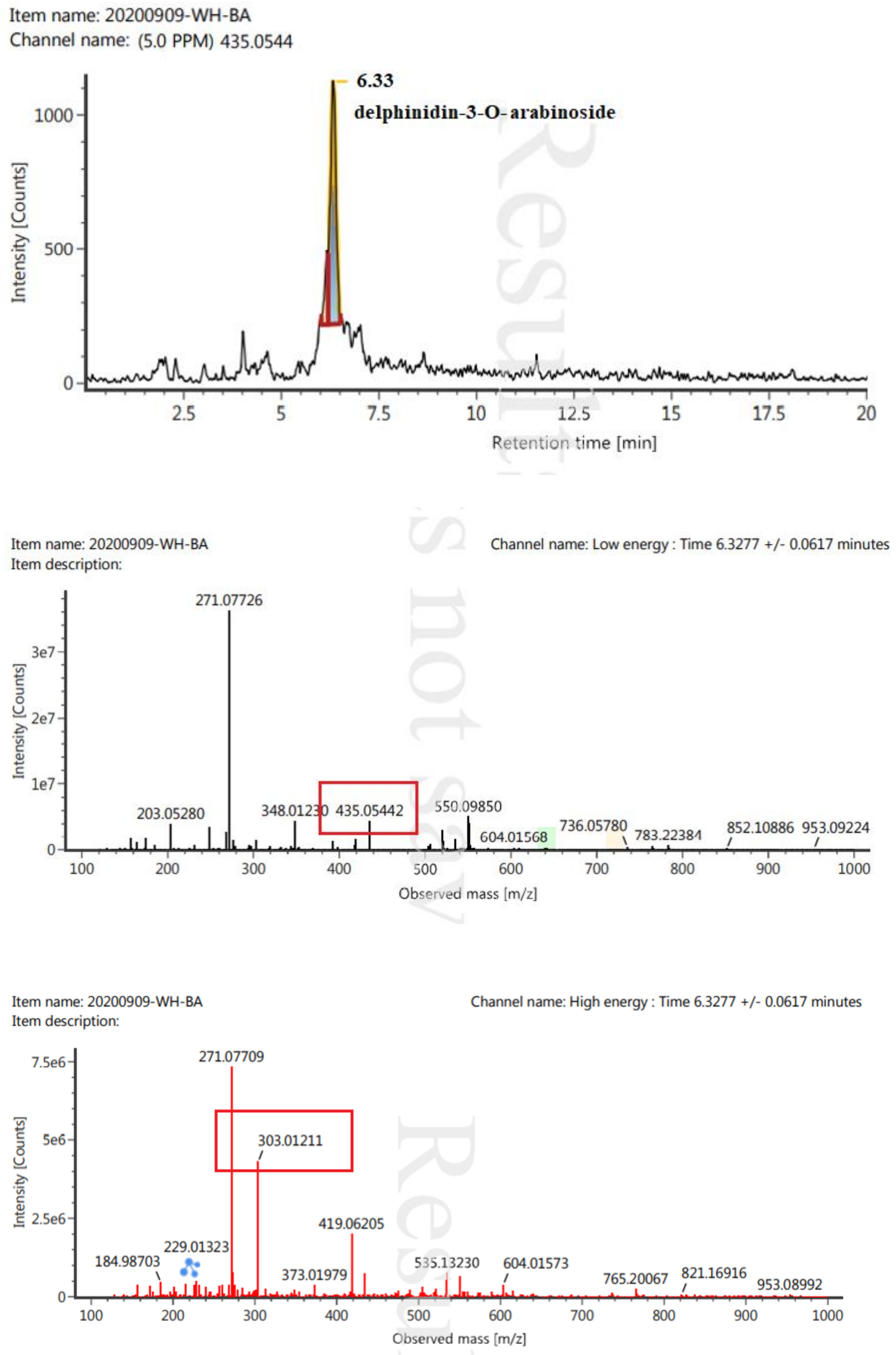

Fig. S6 MS of anthocyanidin compound delphinidin 3-*O*-arabinoside

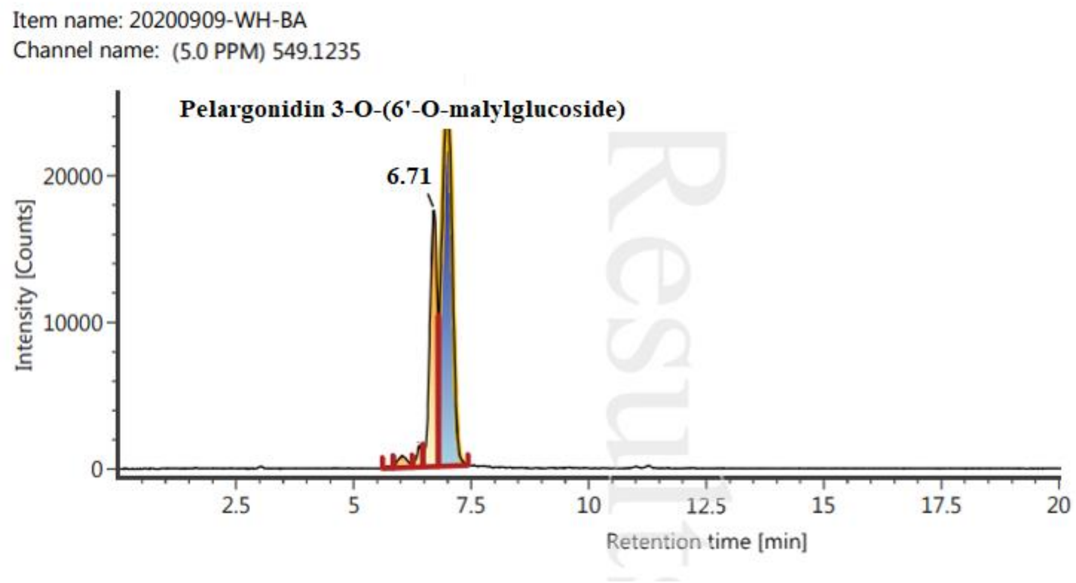

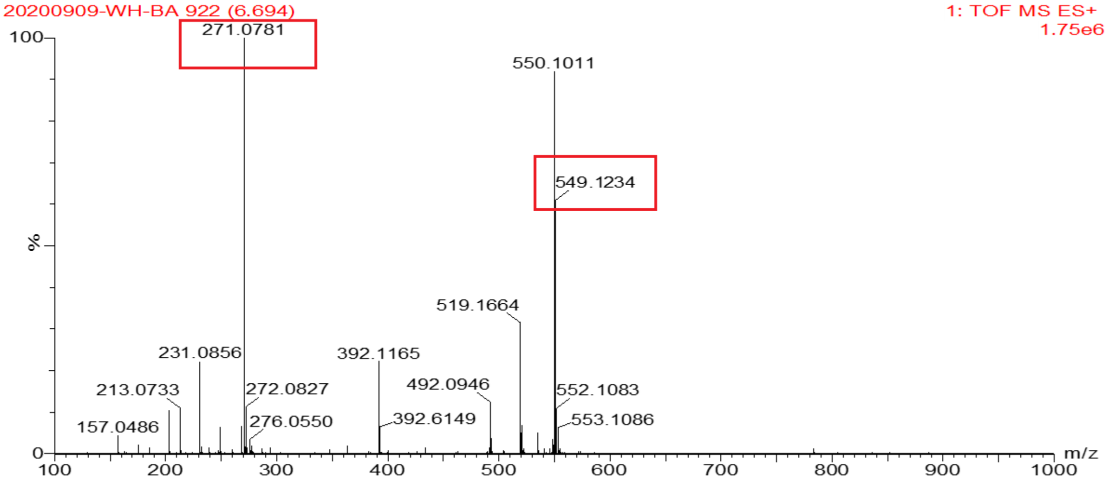

Fig. S7 MS of anthocyanidin compound pelargonidin 3-*O*-(6’-*O*-malylglucoside)

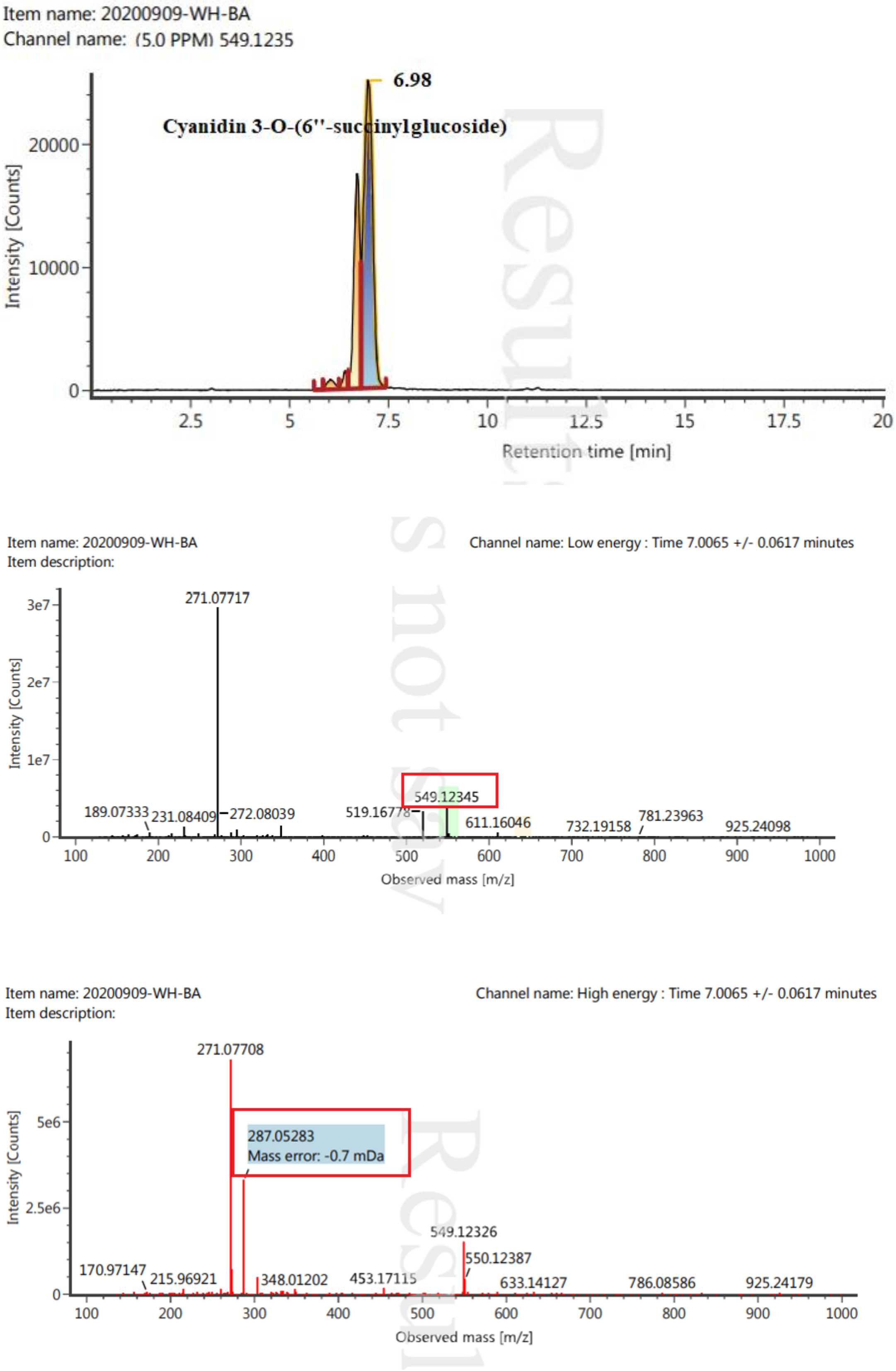

Fig. S8 MS of anthocyanidin compound cyanidin 3-*O*-(6’’-succinylglucoside)

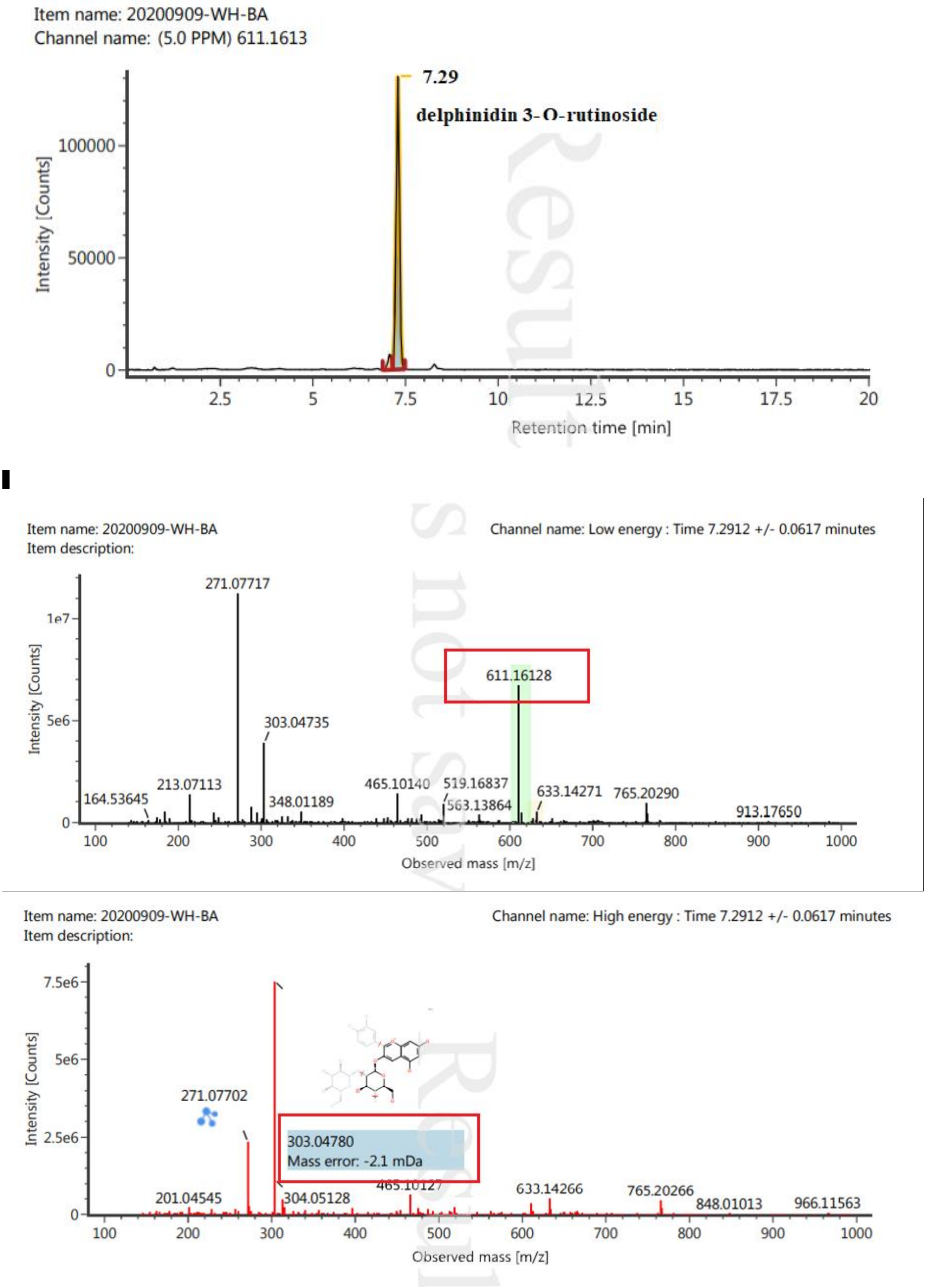

Supplementary Fig. 9 MS of anthocyanidin compound delphinidin 3-*O*-rutinoside

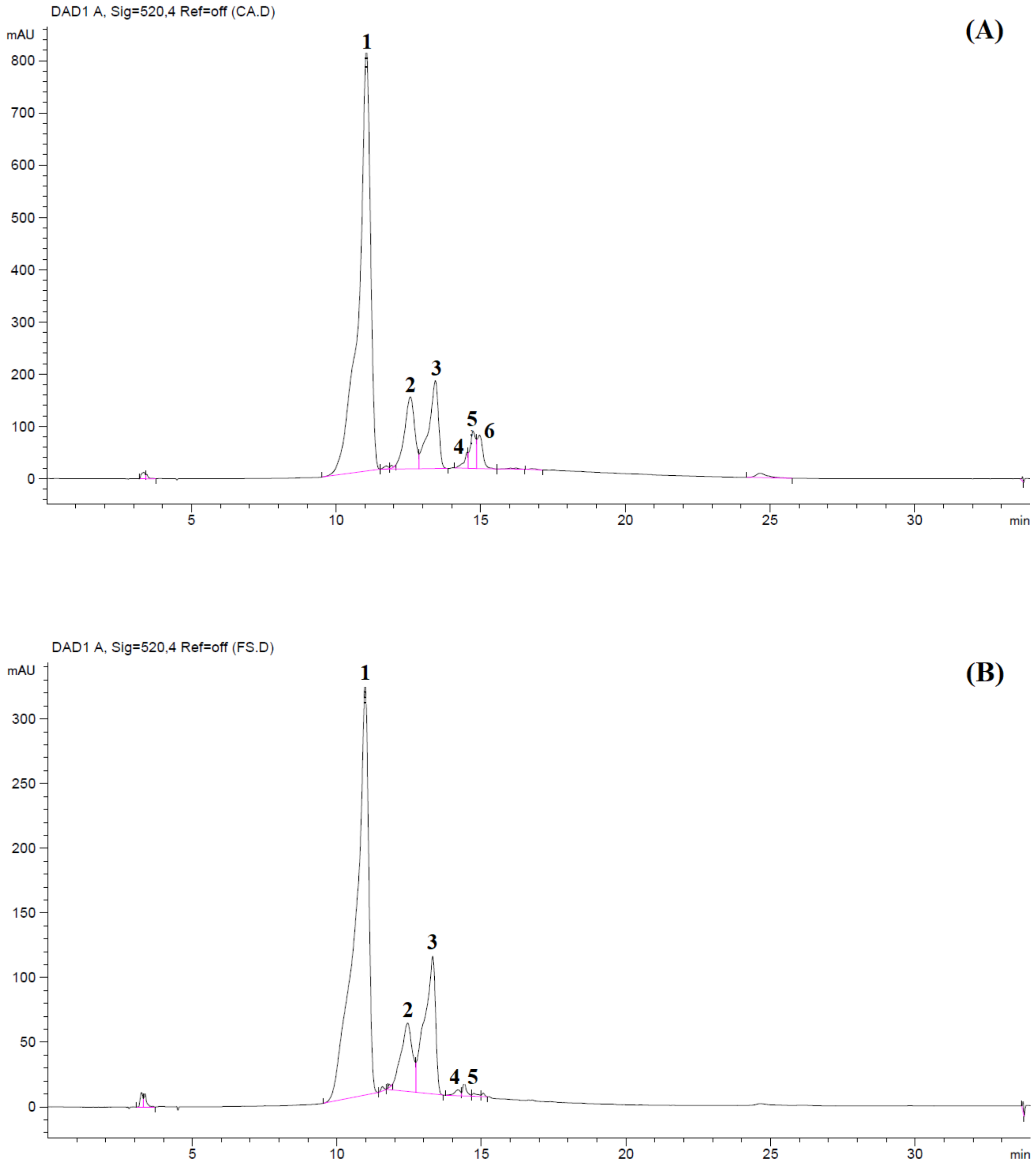

Supplementary Fig. 10 HPLC chromatograms in 520 nm of blackberry crude anthocyanin extracts (BA-CAE) (A) and blackberry fruit slurry extracts (BA-FSE) (B)
