# Supplementary figures and images for: Enhancement on antioxidant, anti-hyperglycemic and antibacterial activities of blackberry anthocyanins by processes optimization involving extraction and purification
Source: Front Nutr. 2022 Oct 11;9:1007691. doi: 10.3389/fnut.2022.1007691 (PMC9593095; doi:10.3389/fnut.2022.1007691)

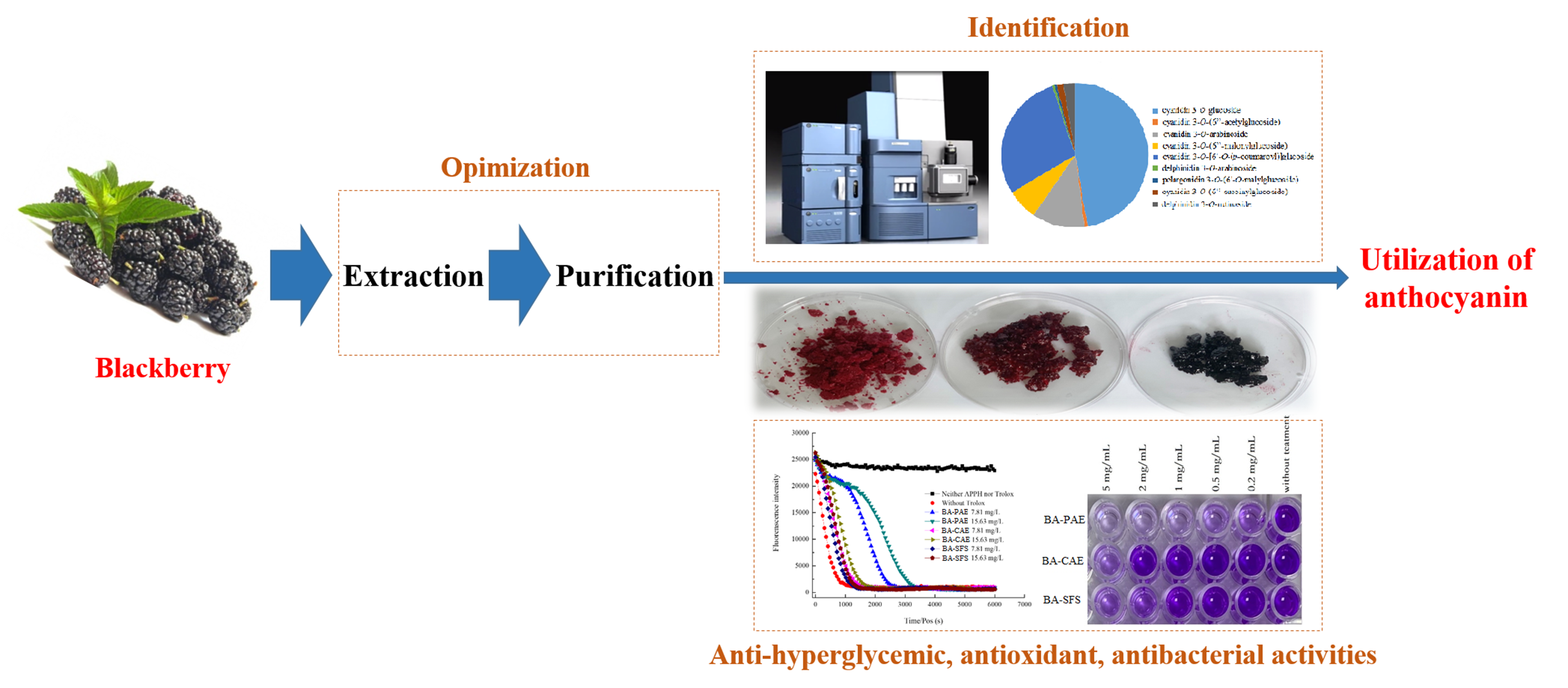

Supplement: Supplementary file 2 [file Image_1.PNG]
